# Characterization of phage vB_EcoS-EE09 infecting *E. coli* DSM613 Isolated from Wastewater Treatment Plant Effluent and Comparative Proteomics of the Infected and Non-Infected Host

**DOI:** 10.3390/microorganisms11112688

**Published:** 2023-11-02

**Authors:** Jimena Barrero-Canosa, Luyao Wang, Angelah Oyugi, Simon Klaes, Pascal Fischer, Lorenz Adrian, Ulrich Szewzyk, Myriel Cooper

**Affiliations:** 1 Institute of Environmental Technology, Chair of Environmental Microbiology, Technische Universität Berlin, Straße des 17. Juni 135, 10623 Berlin, Germany; wangayao11@gmail.com (L.W.); angelah.a.oyugi@campus.tu-berlin.de (A.O.); PascalFischer92@gmx.de (P.F.); ulrich.szewzyk@tu-berlin.de (U.S.); myriel.cooper@tu-berlin.de (M.C.); 2 Institute of Biotechnology, Chair of Geobiotechnology, Technische Universität Berlin, Ackerstraße 76, 13355 Berlin, Germany; simon.klaes@ufz.de (S.K.);; 3 Helmholtz Centre for Environmental Research GmbH—UFZ, Department of Environmental Biotechnology, Permoserstraße 15, 04318 Leipzig, Germany

**Keywords:** coliphage, bacteriophage, proteomics, *Dhillonvirus*, mass spectrometry, LC-MS/MS, stress response, *E. coli*

## Abstract

Phages influence microbial communities, can be applied in phage therapy, or may serve as bioindicators, e.g., in (waste)water management. We here characterized the Escherichia phage vB_EcoS-EE09 isolated from an urban wastewater treatment plant effluent. Phage vB_EcoS-EE09 belongs to the genus *Dhillonvirus*, class *Caudoviricetes*. It has an icosahedral capsid with a long non-contractile tail and a dsDNA genome with an approximate size of 44 kb and a 54.6% GC content. Phage vB_EcoS-EE09 infected 12 out of the 17 *E. coli* strains tested. We identified 16 structural phage proteins, including the major capsid protein, in cell-free lysates by protein mass spectrometry. Comparative proteomics of protein extracts of infected *E. coli* cells revealed that proteins involved in amino acid and protein metabolism were more abundant in infected compared to non-infected cells. Among the proteins involved in the stress response, 74% were less abundant in the infected cultures compared to the non-infected controls, with six proteins showing significant less abundance. Repressing the expression of these proteins may be a phage strategy to evade host defense mechanisms. Our results contribute to diversifying phage collections, identifying structural proteins to enable better reliability in annotating taxonomically related phage genomes, and understanding phage–host interactions at the protein level.

## 1. Introduction

Bacteriophages, viruses that infect prokaryotic cells, are the most abundant entities on earth, with a global estimate of 10^31^ particles [1]. They participate in nutrient cycling through host lysis [2], are drivers of prokaryotic diversity and evolution via horizontal gene transfer (HGT) [3], and can alter host metabolism [4]. Biotechnologically, they have raised renewed interest as a therapeutic alternative to treat infections caused by antibiotic-resistant pathogens (i.e., phage therapy) [5]. Additionally, phage-based strategies are useful in wastewater treatment to reduce pathogen load, bulking bacteria, and biofilm-forming microorganisms [6,7]. Moreover, phages that target enteric bacteria, such as *Escherichia coli*, can serve as surrogates for modeling the fate of enteric viral pathogens in diverse environments [7].

Phages that infect members of *Enterobacteriaceae*, such as *E. coli*, are among the most frequently isolated phages in various environments, including wastewater [8,9,10]. Despite their common and abundant occurrence, only a few representatives, such as phages T4, Lambda, T7, MS2 and M13, and phiX174, have been thoroughly characterized. Yet, their study has led advances in genetics, molecular biology, and virology [11]. Moreover, owing to their resemblance to pathogenic enteric viruses in terms of composition, morphology, and degree of resistance to environmental conditions, they have been proposed as better indicators for fecal pollution than, e.g., *E. coli* [7]. Additionally, virulent phages that infect *E. coli* have been explored for the treatment of intestinal and urinary infections caused by pathogenic and antibiotic-resistant *E. coli* strains. While numerous in vitro studies proved their efficacy (e.g., as reviewed by [12]), clinical trials have not been as successful [13]. Due to their potential application as bioindicators and in phage therapy, understanding their diversity, stability, and interaction with their host cells is required.

Thus far, bacteriophage research has benefited from developing cutting-edge technologies (e.g., all omics technologies). For instance, viral shotgun metagenomics unveiled the diversity and potential ecological function of bacteriophages in all kinds of environments like human and other animals’ digestive tracts, biogas reactors, and marine and freshwater ecosystems [14,15]. However, more than 60% of viral sequences retrieved by viral metagenomics cannot be taxonomically or functionally classified (i.e., viral dark matter) due to the lack of fully characterized viral proteins in databases [16]. Furthermore, viral metagenomes fail to elucidate the complexities of phage–host interactions. 

Alternatively, coupling metagenomics with other omics, such as proteomics, can help clarifying phage–host interactions in model and complex systems [17,18]. In addition, proteomics has facilitated the understanding of complex host–phage interactions beyond the scope of genomic data analysis alone [17]. For example, integrating metagenomics and metaproteomics permitted the functional annotation of unknown proteins of the viral dark matter [18]. Moreover, proteomics coupled with other omics approaches unveiled the link between transcription and translation processes and phage infection efficiencies [19], helped gain insights into phage-mediated host metabolic reprogramming [20], and detected novel phage and bacterial defense mechanisms [21]. Therefore, recent evidence indicates that the combination of phage characterization with proteomics will contribute to a more profound understanding of phage–host interactions.

Thus, while phage research has remarkably grown in recent years, efforts to increase phage collection and perform their comprehensive characterization, including genomic and proteomic analyses, are still needed to advance in microbial ecology and biotechnology. Hence, in this study, we isolated and characterized a phage infecting *Escherichia coli* DSM 613 from the effluent of a sewage plant in Berlin. We analyzed (i) the phage with respect to thermal and pH stability, host range, genomic content, latent period, burst size, morphology, and protein profiles of cell-free lysates, (ii) phage–host interactions by means of differential protein expression of phage-infected and uninfected bacteria, revealing substantial differences between infected and uninfected cultures in amino acid and nucleotide metabolism, aligning with the anticipated metabolic interdependence of the phage and its host for virion synthesis.

## 2. Materials and Methods

### 2.1. Host Strain

*Escherichia coli* DSM 613 was maintained in LB medium (10% (*w*/*v*) peptone, 5% (*w*/*v*) yeast extract, 10% (*w*/*v*) NaCl) and grown at 37 °C with constant shaking at 110 rpm. 

### 2.2. Collection of Samples for Bacteriophage Isolation

Surface water was collected from the river Erpe in Berlin after the discharge of the wastewater treatment plant “Münchehofe” (52°29′3.80″ N; 13°38′43.30″ E; Supplementary Figure S1). The samples were kept at 4 °C upon processing. To remove particles and unicellular microorganisms, the water was centrifuged for 10 min at 10,000× *g* and then serially filtered with 0.45 µm and 0.2 µm pore size PVDF (Durapore) filters.

### 2.3. Phage Enrichment and Isolation

Phage enrichment was carried out as described previously [22]. Briefly, 10 mL of LB medium with double-strength concentration was mixed with 10 mL of the 0.2 µm filtered water and inoculated with 100 µL of an *E. coli* overnight culture. The mix was incubated at 37 °C. After 24 h of incubation, the culture was centrifuged at 10,000× *g* for 10 min. Thereafter, the supernatant was recovered, and 500 µL of chloroform was added. The supernatant was serially diluted 1:10 until a concentration of 10^−7^ was reached. Next, this dilution was used for the isolation of the phage using the double agar overlay assay [23]. Clear single plaques were individually collected in tubes and resuspended in LB medium before the agar overlay was repeated. The phage isolate was considered pure after three consecutive repetitions.

### 2.4. Thermal and pH Stability Analysis

For the thermal stability tests, 1 mL of the phage suspension was incubated at 4, 22, 30, and 37 °C for 24 h. For each suspension, the titer was calculated before and after incubation using the agar overlay assay. For the pH stability analysis, 1:10 phage suspensions were prepared with sterile SMG buffer (100 mM NaCl; 8.1 mM MgSO_4_; 50 mM Tris 0.01% (*w*/*v*) gelatin) and adjusted to pH 3, 5, 7, 9, and 10. The mixtures were incubated at room temperature for 24 h. The titer of each suspension was determined before and after incubation via the agar overlay assay. All assays were performed in triplicate.

### 2.5. Host Range Analysis 

Host range tests were conducted using spot testing [24] and using the following 17 *E. coli* strains as host: BL 21, DH5 alpha (meth deletion), DSM 1103, DSM 5695, DSM 613, DSM 4230, HB101 (pRK2013,Km), HS (meth deletion), HS996 (meth deletion), JM 101, JM 109, K12, MG1655, pcK218:jim4, S17-1 lambda pir, SM10 lambda pir (pUX-BF13, Ap), and VH33 (Supplementary Table S1). All strains were plated using the agar overlay technique, and the phage lysates were diluted 10-fold, obtaining concentrations ranging from 10^0^ to 10^−10^. Next, 10 µL of each dilution was spotted onto each bacterial overlay. The plates were incubated at 37 °C overnight. The presence of clear spots and the highest dilution at which lysis was observed determined the classification of phage infectivity.

### 2.6. Genome Sequencing and Analysis

Phage DNA was extracted from cell-free lysates. Prior to DNA extraction, phages were concentrated using PEG 8000 as previously described [25]. To remove host DNA and RNA, the samples were treated with 41 Kunitz Units of DNAse I (Qiagen) and with 0.07 mg mL^−1^ of RNAse A (Thermo Fisher Scientific, Waltham, MA, USA). DNA was extracted with a phage DNA isolation kit (Norgen Biotek Corp., Thorold, ON, Canada) following the manufacturer’s instructions. The genome was sequenced on the Illumina NovaSeq platform (paired-end reads). For the genome de novo assembly, the quality of Illumina reads was improved using BayesHammer [26]. Error-corrected reads were assembled using SPAdes v. 3.10 [27], scaffolding was performed using SSPACE version 2.3 [28], gapped regions within scaffolds were partially closed using GapFiller version 1.10 [29], and assembly errors were corrected using Pilon version 1.21 [30]. Next, PhageTerm (Galaxy V 1.0.12) was used to reorder the genome based on the phage termini and packaging prediction [31]. The genome was annotated using two strategies: (1) with MultiPhATE [32], using PHANOTATE as a gene caller [33] and, to annotate ORF calls, PhAnToMe (included in the MultiPhATE tool from https://github.com/carolzhou/multiPhATE accessed on 9 June 2020), pVOGs [34], and SwissProt [35]; (2) with the Rast webserver [36], using the RASTtk annotation scheme [37] and glimmer3 [38] and prodigal [39] as gene callers. A consensus annotation was created and manually curated using Snapgene v. 5.1.3 (from Insightful Science; available at snapgene.com). The linear genome map was visualized with Snapgene v. 7.0.2. 

To predict the phage lifestyle, Phage Classification Tool Set PHACTS and PhaTYP were used [40,41]. PHACTS uses a similarity algorithm and a supervised Random Forest classifier to predict whether a phage is temperate or virulent [40]. PhaTYP uses the machine learning model “Bidirectional Encoder Representations from Transformer” (BERT) and protein-based tokens for phage lifestyle prediction [41]. To establish the relatedness of the isolated phage to known phages, the draft genome was blasted against the NCBI nt database, and sequences that produced a significant alignment (i.e., E-value 0.0; >80% nucleotide identity; >75% query coverage) were downloaded and used to calculate the intergenomic distance with VIRIDIC, using a species threshold of 95%, a genus threshold of 70%, and default settings [42]. Additionally, to establish the phylogenomic relatedness to phage species from the NCBI database that produced a significant alignment, a hierarchical tree was constructed with VirClust-based protein clustering (PC) with 1000 bootstraps [43]. Moreover, a network-based whole-genome gene sharing profile was created with vConTACT2 (v. 0.9.22) using the Prokaryotic ViralRefSeq v94 database [44]. The clustering analysis was visualized with Cytoscape v. 3.9.1. [45]

### 2.7. One-Step Growth Curve Experiments 

To assess the latent period, rise, and burst size of phage EE09, a one-step growth curve was determined as described elsewhere [46]. *E. coli* cells were grown in LB medium supplemented with 5 mM MgSO_4_ and with a multiplicity of infection, MOI, of 0.001 and 5. Burst size was calculated as the ratio of the final count of free phage particles to the initial count during the latent period. 

### 2.8. Transmission Electron Microscopy (TEM) 

To determine the phage morphology, 2 mL of a lysate with a 10^11^ PFU mL^−1^ was centrifuged at 25,000× *g* for one hour. The pellet was washed twice and resuspended in a 0.1 M ammonium acetate solution (pH 7.0). The viral particles were stained with 1% (*w*/*v*) uranyl acetate for 10–20 s [47]. TEM was conducted with a BioTwin CM 120 microscope (Philips; operated at 80 V). The dimensions of the virions were measured on micrographs with Analysis Pro (iTEM) software v. 2.11.

### 2.9. Protein Analysis 

To detect structural proteins of the phage EE09, 25 mL of cell-free high-titer phage lysate was filtered using 0.2 µm pore size PVDF (Durapore) filters. Next, the virions in the filtrate were harvested by ultracentrifugation at 82,000× *g* for 2 h at 4 °C, the supernatant was discarded, and the samples were resuspended in 100 µL of native lysis buffer and kept overnight at 4 °C. The samples were disrupted by five cycles of freezing in liquid nitrogen and thawing in a thermal shaker at 40 °C and shaken at 750 rpm for 2 min. The disrupted cells were sonicated for 30 s in an ultrasonic bath and cell debris was removed by centrifugation at 16,000× *g* for 10 min at 4 °C. The protein concentration of the supernatant was estimated using a DS-11 µVolume spectrophotometer (DeNovix) at 280 nm using the native lysis buffer as the blank. As the internal standard, 2 µL of the BSA standard was added to a final concentration of 80 ng of BSA per sample. A 10% DOC stock solution was added to the sample to a final concentration of 5% (*w*/*v*). This resulted in the formation of a white gel suspension. Protein reduction, alkylation, and protein digestion were conducted with 12 mM dithiothreitol (DTT) at 37 °C, slightly shaking for 30 min, 40 mM 2-iodoacetamide (IAA) for 45 min at room temperature in the dark, and 0.63 µg of trypsin (sequencing grade), respectively, as described in [48]. Formic acid was added to a final concentration of 2.5% (*v*/*v*) to stop the digestion. Undigested and precipitated proteins were removed by centrifugation (16 000× *g*, 10 min, 4 °C). Desalting was carried out with Pierce^®^ C18 Tips (Thermo Fisher Scientific, Waltham, MA, USA), following the manufacturer’s instructions with modifications. The peptides were subsequently dried by vacuum centrifugation and reconstituted with 0.1% (*v*/*v*) formic acid. The peptides were analyzed using a nano-liquid chromatographer coupled to a tandem mass spectrometer (Thermo Orbitrap Fusion, Thermo Fisher Scientific, Waltham, MA, USA).

To compare the protein expression of infected vs. uninfected cells, we established a protocol for protein extraction based on previously published setups, with some modifications [17,19,49]. Briefly, 50 mL of LB medium was inoculated with 70 µL of an overnight culture and incubated at 37 °C until the culture reached an OD_600nm_ of 0.2. Then, the cultures were equally divided into two new sterile flasks, with approx. 25 mL in each. One culture was infected with the phage EE09 with MOI of 5, and the second culture was mock-infected with an equivalent volume of SMG buffer. After 25 min of infection, 2 mL of each culture was collected and centrifuged at 21,910× *g* for 2 min. Then, the pellets were washed twice with 100 mM Ambic buffer pH 7.9, immediately frozen in liquid nitrogen, and stored at −80 °C until further processing. For protein extraction, the cells were disrupted using 5 cycles of freezing and thawing as described above. Protein reduction, alkylation, protein digestion, and desalting were performed as described above for the cell-free lysate, and a detailed protocol, including downstream analysis, is reported in the Supplementary method Section. The peptides were analyzed using nano-liquid chromatography coupled to tandem mass spectrometry (Thermo Orbitrap Fusion, Thermo Fisher Scientific, Waltham, MA, USA) [50]. All experiments were carried out in biological triplicates.

### 2.10. Analysis of the Proteomic Data 

The mass spectrometric raw data were analyzed with Proteome Discoverer v2.4 (Thermo Fisher Scientific). The spectra were searched against a custom database that included the *E. coli* DSM 613 protein database retrieved from the ATCC Genome portal (https://genomes.atcc.org/; accessed on 30 August 2023; culture col. no.: ATCC 11303 in the American Type Culture Collection, equivalent to DSM 613 of the German collection), common laboratory contaminants, and the proteins of phage EE09 annotated as described above, totaling 4173 entries (Data Set S1). Spectra searches were carried out with SequestHT implemented in Proteome Discoverer v 2.4 using the following settings: mass tolerance ±3 ppm and ±0.1 Da for precursor and fragment ions, respectively, peptide length restricted to 6–144 amino acids and up to two missed trypsin cleavages, dynamic oxidation of methionine, static carbamidomethylation of cysteine, and false discovery rate <0.01 at peptide and protein level using a target decoy approach. The label-free proteins were quantified using the Minora Feature Detector from the Proteome Discoverer Software. Intensities were normalized between samples using total peptide amounts, and *p*-values for protein ratios were calculated by background-based t-test and adjusted by Benjamini–Hochberg correction for the false discovery rate (q-values). Proteins were filtered based on the following criteria: detection in at least two replicates (for changes in protein abundance between infected and control cultures, we considered only proteins that were found “high” in two infected replicates as well as in two control replicates), >1 peptide, and high FDR confidence. Changes in protein abundances were plotted on a volcano plot using VolcaNoseR [51]. The cutoff value log2 (abundance ratio) of ±1 and *p*-value (adjusted) <0.05 were used to identify the significantly differentially expressed proteins.

## 3. Results and Discussion 

### 3.1. Characterization of Escherichia Phage vB_EcoS-EE09

**Figure 1:**
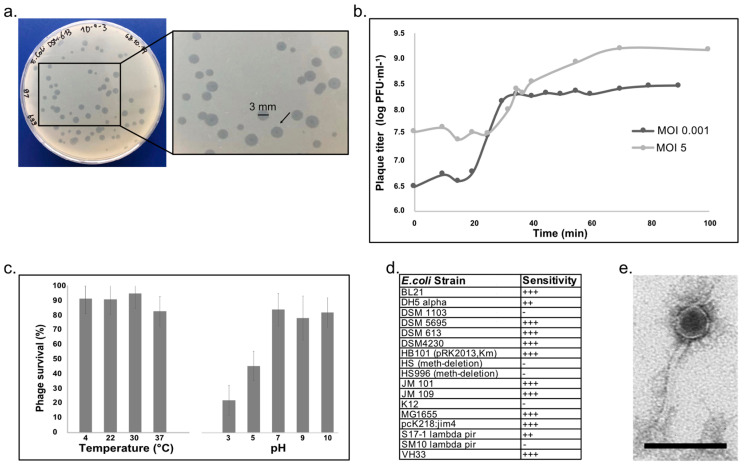
Cultivation-based characterization of phage EE09. (**a**) Plaque assay of phage EE09 in soft agar medium, black arrow pointing to bull-eye morphology. (**b**) One-step growth curves of EE09 in LB medium using two different multiplicity of infection (MOI) values, i.e., MOI 0.001 (dark grey) and MOI 5 (light grey). (**c**) Stability tests carried out for 24 h in triplicate at different temperatures (4, 22, 30, 37 °C) and pH (3, 5, 7, 9, 10). Data show means of triplicate tests, and error bars show SD. (**d**) EE09 host range tested using 17 *E. coli* strains; +++ refers to strains that were highly sensitive, i.e., clear lysis was observed, at a dilution higher than 10^−6^, ++ refers to strains for which turbid lysis was observed or lysis was observed only at a dilution of 10^−5^, - refers to strains resistant to EE09, as no lysis was observed at any dilution. (**e**) Transmission electron micrograph showing the morphology of EE09 virion particles. Scale bar: 100 nm.

Escherichia phage vB_EcoS-EE09 was isolated from an enrichment culture prepared with water collected from the effluent of the wastewater treatment plant (WWTP) “Münchehofe” entering the river Erpe (Supplementary Figure S1). For simplification, we will refer to phage vB_EcoS-EE09 as phage EE09 in the rest of the text. In vitro, phage EE09 produced bull eye-like lytic plaques of a diameter of approximately 3–4 mm, and sporadically, smaller plaques of <1 mm were observed on double-agar LB plates supplemented with 5 mM MgSO_4_ (Figure 1a). Moreover, a one-step growth curve experiment was carried out to determine EE09 latent period and burst size. When *E. coli* was grown in LB and infected with EE09 at a multiplicity of infection, MOI, of 0.001, the latent period lasted 20 min, with a 10 min rise period and a burst after 30 min of infection, with an average burst size of approximately 93 phages per infected cell (Figure 1b). When *E. coli* was grown in LB and infected at an MOI of 5, the latent period lasted 25 min, with a 45 min rise in the titer and a full burst after 70 min of infection. For an MOI of 5, the average burst size was 43 phages per infected cell (Figure 1b). The decrease in the burst size may be attributed to different factors: (i) a high titer of phage EE09 compared to the number of the available host cells, which could induce cell lysis externally from the host without a successful infection (referred to as “lysis from without”) [52], (ii) an increase in the infection kinetics that could lead to cell lysis prior to the assembly of all phage progeny [53], or (iii) the phage entering a lysogenic state due to a high phage-to-bacterial cell ratio [54]. The lifestyle predictions of EE09 based on genomic analysis with two different strategies, PHACTS and PhaTYP, presented a contradictory outcome. Specifically, the PHACTS analysis predicted a temperate lifestyle (with a probability of 0.514 ± 0.043), whereas PhaTYP predicted a virulent lifestyle with a higher confidence (score 0.99). These contradictory results underscore the significance of the ongoing in vitro characterization of phages. Such efforts are crucial for reducing uncertainty in predictive outcomes. Furthermore, the thermal and pH stability of phage EE09 was assessed at different temperatures (i.e., 4, 22, 30, and 37 °C) and pH values (i.e., 3, 5, 7, 9, and 10). EE09 was stable at 4, 22, and 30 °C. The phage titer was reduced by 17.5% when the phage was incubated at 37 °C (Figure 1c). Regarding the pH, EE09 was more stable in conditions from neutral to basic and lost approximately 70% and 50% of infectivity at pH 3 and pH 5, respectively (Figure 1c). Thus, EE09 appeared more stable under basic than under acidic conditions. Additionally, we evaluated the host range of EE09 by testing its infectivity in 17 *E. coli* strains. EE09 infected and lysed 12 of the 17 strains tested, showing that the phage is not restricted to a specific *E. coli* strain (Figure 1d). 

**Figure 2:**
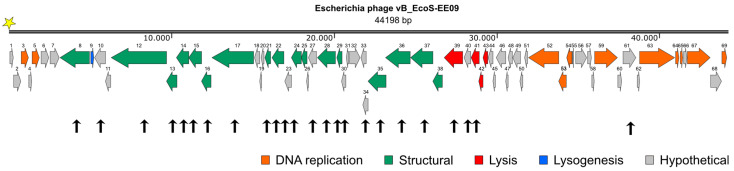
Graphic representation of the linear genome of Escherichia phage vB_EcoS-EE09. Encoded proteins are represented in colors based on their annotated function category. Numbers indicate the CDS. Orange: proteins involved in DNA replication; green: structural proteins; red: proteins involved in cell lysis; blue: proteins involved in DNA recombination/lysogenesis; grey: hypothetical proteins. Yellow star indicates the 5′ cohesive end (ATCTTAAGGG) predicted using PhageTerm (Galaxy V 1.0.12) [31]. Black arrows indicate proteins detected in cell-free phage lysates using Thermo Orbitrap Fusion LC-MS/MS.

Phage EE09 virions had a capsid of approx. 46 nm and a flexible tail of approx. 107 × 11 nm (Figure 1e). Thus, based on its morphology, we classified EE09 as member of the class *Caudoviricetes*. This classification was corroborated and complemented after genome sequencing and further phylogenetic clustering analyses with VirClust [43] and vContact 2.0 [44] (Figure 2 and Supplementary Figure S2 and Figure S3, respectively). Moreover, based on the genome species demarcation criterion of 95% [55] and our calculations of intergenomic similarities at the nucleic acid level of phage EE09 with VIRIDIC [42], phage EE09 was identified as a novel species in the genus *Dhillonvirus* (Supplementary Figure S4). In this genus, EE09′s closest relatives are Escherichia phage GeorgBuechner (identity: 91.90%, query coverage: 96%), Escherichia phage SECphi18 (identity: 94.23%, query coverage: 96%), and Escherichia phage welsh (identity: 93.95%, query coverage: 94%) (Supplementary Table S2). Members of *Dhillonvirus* were isolated from other wastewater environments [8,9]. However, even though multiple phages belonging to the genus *Dhillonvirus* were isolated and their genomes sequenced [10], little to no information regarding phage–host interactions or in vitro characterization is available. 

Phage EE09 has a dsDNA linear genome of approx. 44,198 bp (97% completeness) with 54.6% GC content (Figure 2; deposited in the NCBI database under the accession number OR756193). Statistical details of the genome sequencing are described in Supplementary Table S3. Using PhageTerm, we predicted that phage EE09 possesses a cohesive end in the 5′ end (COS 5′) of ten nucleotides in length with the sequence ATCTTAAGGG [31] (Supplementary Figure S5). COS 5′ ends are also present in phage Lambda and are recognized by the phage terminase (CDS37 and CDS38; Supplementary Table S3). The phage terminase cleaves the DNA to be packed in the viral particles leaving cohesive endings [56]. Once the phage has infected a new host cell, phage EE09 circularizes through its cohesive ends, like other phages with 5’ COS endings. This circularization is a vital step in the replication cycle.

The EE09 genome was arranged in functional modules, grouping genes encoding for proteins involved in genome replication, structural conformation of viral particles (structural genes), cell lysis, and lysogenesis, as performed for other phages (Figure 2) [21]. We identified 69 coding sequences (CDS) and predicted 11 of them to encode proteins involved in phage replication (e.g., DNA polymerases), 17 to encode structural proteins (e.g., major capsid, tail proteins), 4 to be involved in host lysis (e.g., holins and lysins), 1 to participate in lysogenesis (e.g., super-infection exclusion protein). We also identified 41 genes encoding proteins without predicted function, which were annotated as hypothetical proteins (Figure 2). No tRNA-encoding gene was identified. The genome size and organization agreed with the description of other *Dhillonvirus* phages [8]. Detailed characteristics and annotation of the EE09 genome are provided in Supplementary Table S4.

### 3.2. Proteomic Analysis of Cell-Free Lysates of Escherichia Phage EE09

Cell-free phage lysates were analyzed to identify structural proteins of EE09 viral particles. We identified 24 phage proteins in the lysates, with sequence coverage ranging from 3 to 80% (Figure 2 and Table 1). Among them, 16 were annotated as structural proteins, representing 94% of the 17 encoded proteins identified as structural proteins. Two proteins detected appeared to be involved in cell lysis (CDS40, CDS41). Nine hypothetical proteins were detected (CDS10, CDS23, CDS27, CDS30, CDS34, CDS40, and CDS61). Given the high functional modularity of phage EE09 genome, it is very likely that CDS10, CDS23, CDS27, CDS30, and CDS34, are structural proteins, like other proteins encoded within the genomic region from CDS8 to CDS36 (region of structural proteins’ location in phage EE09 genome; Figure 2; Supplementary Table S4).

CDS40 and CDS41 are likely involved in cell lysis. CDS40 encodes a lysin. Their presence in the lysate can be attributed to two possibilities: (i) these proteins may be associated with the tail fibers of the virion particles, potentially facilitating host cell wall breakdown upon infection, as observed for other phages (reviewed in [57]), (ii) they might have been detected in the lysate because they were expressed just before host cell lysis, which allowed them to pass through the 0.2 µm pore size filter during the preparation of the cell-free lysates. The latter explanation also accounts for the presence of CDS37, a phage terminase, and CDS39, a putative phosphodiesterase, in the lysates. The presence of CDS61, a hypothetical protein within the replication module (Figure 2), was somewhat unexpected (Figure 2). CDS61 may have persisted in the lysate due to its higher stability compared to that of other proteins, possibly as a remnant from prior host infections.

Many of the structural proteins, such as tail and capsid components, exhibited a good sequence coverage (Table 1), with CDS29 (capsid and scaffold protein) showing the highest sequence coverage of 80%. Thus, the identified peptides within the proteome of the phage lysates further supported the gene annotations delineated within the EE09 genome, and our proteomic strategy was efficient for detecting virion proteins. 

### 3.3. Detection of Escherichia Phage EE09 Proteins in Infected E. coli Cultures 

After validating protein detection in cell-free phage lysates, we tested if phage proteins could be detected in *E. coli* cultures infected with phage EE09. To this end, we grew *E. coli* in LB medium and infected it with phage EE09 during the mid-log phase, at an MOI of 5. We collected samples of the infected cultures as well as of the non-infected controls after 25 min of infection, which marked the end of the phage eclipse period preceding cell lysis (Figure 1b). 

In the infected cultures, we detected only four phage proteins (CDS29, CDS40, CDS56, and CDS61). Notably, CDS29 was the sole putative structural protein detected in the infected cultures. The other three proteins, CDS40, CDS56, and CDS61, are hypothetical proteins. Finding only one structural protein was unexpected, given that the time of sample collection was near cell lysis. The low detection of structural protein may be attributed to (i) biological or (ii) technical reasons: (i) it is possible that the phage underwent a lysogenic or pseudolysogenic cycle and, therefore, the lytic cycle was halted, with the consequence that few structural proteins were found, (ii) phage proteins were lost during processing due to the higher abundance of host proteins in the cultures. In the latter case, performing SDS-PAGE gels for protein separation and size-fractionated mass spectrometry may improve the detection of phage proteins [58]. Phage proteins were not detected in the non-infected controls. 

### 3.4. Comparative Proteomic Analysis between E. coli Infected with Phage EE09 and Non-Infected Cells

To elucidate the impact of EE09 infection in *E. coli*, we compared the full proteome of *E. coli* cells grown in LB medium when infected with phage EE09 at an MOI of 5 to that of non-infected controls using a single time point right before the end of the eclipse phase (i.e., 25 min post infection). All experiments were carried out in triplicate.

Using nLC-MS/MS, a total of 1176 bacterial proteins were detected after filtering (Section 2.10), representing ~28.37% of our *E. coli* protein database. Among these, 1060 were present in both infected cultures and non-infected controls, 48 were exclusively detected in the infected cultures, and 68 were only found in the non-infected controls. To assign functional categories, all bacterial proteins detected were queried against the KEGG database using BlastKOALA (Supplementary Figure S6) [59]. Next, we organized all detected proteins into functional categories and estimated the relative abundances per functional category. The relative abundances of the proteins detected in infected and control cultures were similar; of these, ~56.6% were found to be involved in genetic information processing, ~37.5% in metabolic processes, ~2.7% in signaling and cellular processes, and ~1% in environmental information and processing, while ~2.5% were unclassified (Figure S7).

Next, we evaluated if any protein detected in both infected cultures and non-infected controls was significantly differentially expressed by comparing the log2 of the fold change in protein abundance between infected cultures and non-infected controls. Most of the host proteins, 1041, were not significantly differently expressed. The small differences between infected and non-infected cultures at the host proteome level could be due to the selected time point. According to the half-life of a protein, changes can be induced by dilution due to cell division or degradation [60]. Therefore, changes in the abundance of proteins with a half-life longer than 25 min might have been very small in our experiments. In addition, no significant differences in the overall proteome between infected and non-infected host cells may suggest that significant differences at the protein abundance level would only be detectable when cell lysis starts, as observed for the coliphage phiX174 [17], or that significant changes could be more pronounced at the transcription level [20]. Alternatively, no significant differences were observed in the host proteome of phi38:1 phage, a generalist phage that can infect multiple *Cellulophaga baltica* strains [19].Phage EE09 is also a generalist phage, as it infects various *E. coli* strains (Section 3.1), suggesting that such a trait might be shared among generalist phages. However, this hypothesis is currently weak due to the limited number of relevant studies. Further research, ideally with a more time-resolved analysis of the protein abundance of multiple generalist phages, would be necessary to corroborate these observations.

**Figure 3:**
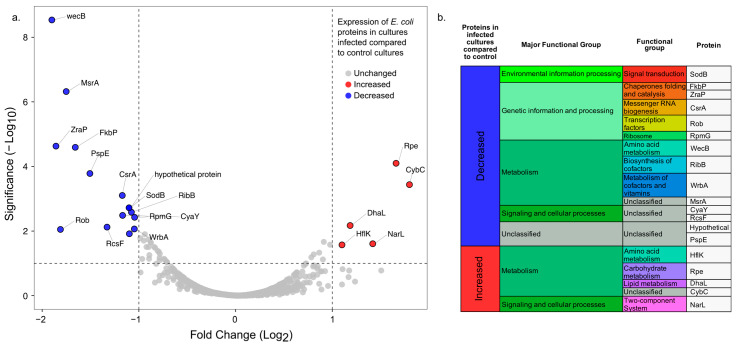
Differential abundance of *E. coli* proteins after 25 min of phage EE09 infection compared to controls (mock-infected with SMG buffer). (**a**) Volcano scatter plot showing all proteins detected in *E. coli* cultures using FC ± 1 and *p* value < 0.05 as a significance threshold; in gray, all proteins whose expression was not statistically significant, in blue, proteins that were significantly less abundant in EE09-infected cultures compared to the controls, and in red, proteins that were significantly more abundant in the infected cultures. Data were plotted in VolcaNoseR web app [51]. (**b**) Table showing the 19 significantly differentially expressed proteins and their respective functional category based on BRITE functional hierarchies [59].

We detected 19 proteins that were significantly differentially expressed (*p*-value < 0.05; FC ± 1), of which, 5 showed a higher relative abundance, and 14 showed a lower relative abundance in the infected cultures compared to the non-infected controls (Figure 3a). Proteins with a higher relative abundance in the infected cultures were annotated as being involved in either metabolic functions or signaling and cellular processes (Figure 3b). These proteins were ribulose phosphate-3-epimerase (*rpe*), nitrate/nitrite response regulator protein NarL (*narL*), dihydroxyacetone kinase L subunit (*dhaL*), HflK protein (*hflK*), and soluble cytochrome b 562 (*cybC*) (Figure 3). 

The HflK protein exhibited a significantly higher abundance in the infected cultures than in the controls (Figure 3). HflK is a membrane protein that forms a complex with the HflC protein, and the complex has protease activity [61]. The higher expression of HflK in infected cells compared to the controls is particularly intriguing because this protein is involved in the establishment of lysogeny in the Lambda phage [62]. Interestingly, the gene for a detected phage protein (CDS56; hypothetical protein; Section 3.3) is near a gene that putatively codes for a transposase (Figure 2; Supplementary Table S4). Given the highly modular nature of phage genomes, it is plausible that CDS56 plays a role in DNA recombination processes. Thus considering (i) the higher relative abundance of HflK in infected cells compared to the controls, (ii) the detection of the CDS56 phage protein, (iii) the absence of structural proteins in the proteome before the onset of lysis, and (iv) the observed decreased burst size with high MOI (i.e., MOI 5, Section 3.1; Figure 1b) [54,63,64], EE09 is likely a temperate phage. However, the phage lifestyle predictions from the PHACTS and PhaTYP tools yielded contradictory results, hindering a definitive conclusion. Thus, further experiments are needed to confirm EE09 lifestyle. 

**Figure 4:**
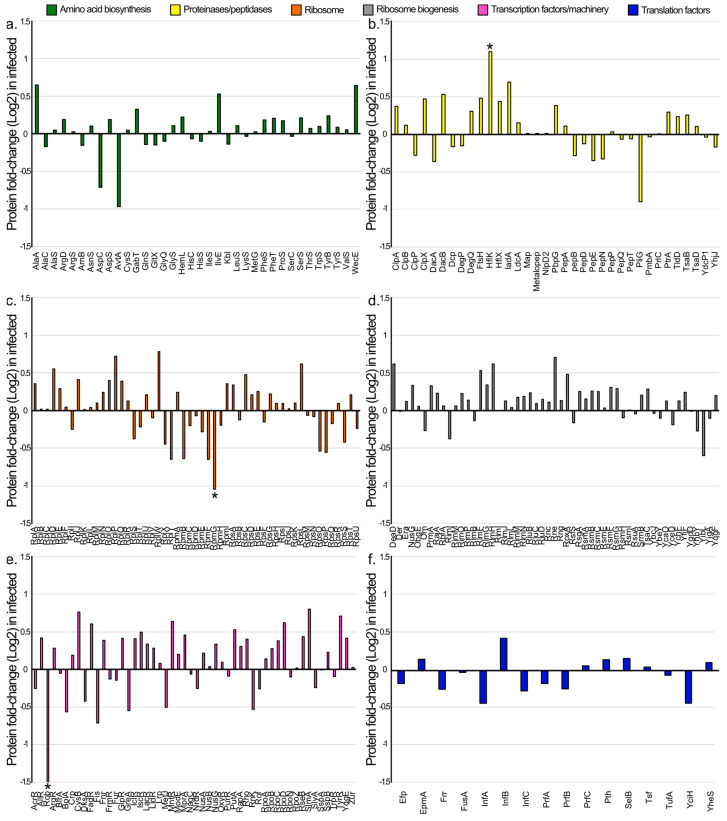
Changes in the abundances of the 244 proteins involved in the synthesis and degradation of proteins in the infected cultures compared to the non-infected controls, expressed as Log2 of the ratio of the abundance in infected cells to the abundance in the controls. (**a**) Amino acid biosynthesis (36 proteins). (**b**) Peptidases/proteases (35 proteins). (**c**) Ribosomal proteins (53 proteins). (**d**) Ribosome biogenesis (53 proteins). (**e**) Transcription factors and machinery (51 proteins). (**f**) Translation factors (16 proteins). Marked with asterisks are proteins whose fold change was significant (*p*-value < 0.05; FC ± 1).

Alternatively, the overexpression of HflK could be attributed to other factors. For instance, since HflK is a protease, phage EE09 might increase its expression to recycle bacterial proteins for synthesizing its own proteins. In line with this hypothesis, we examined the fold-change values of all proteins involved in the synthesis and degradation of proteins (Figure 4). We observed that 64% of these proteins were more abundant in EE09-infected cultures. Moreover, this trend was more marked for proteins involved in amino acid biosynthesis (67% slightly more abundant than in the non-infected controls; Figure 4a), peptidases/proteases (60% substantially more abundant than in the non-infected controls; Figure 4b), ribosomal proteins (58% slightly more abundant than in the controls; Figure 4c), ribosome biogenesis (72% slightly more abundant than in the controls; Figure 4d), and transcription and transcription machinery (69% substantially more abundant than in the non-infected controls; Figure 4e). In addition, the proteins involved in translation were the only ones to exhibit an opposite trend, as 66% of them showed a lower relative abundance in infected cells compared to the controls (Figure 4f). As reported, a reduction in proteins involved in bacterial translation is likely due to the phage modulating the cellular machinery to prioritize its own protein synthesis, while inhibiting cellular processes [65]. Overall, these results confirmed that the phage EE09 influenced the host to promote the synthesis of its own proteins. Furthermore, the absence of significant differences in most of these proteins may be attributed to the specific experimental conditions used. In a nutrient-rich medium like LB and during the log growth phase, it is plausible that essential building blocks required for virion synthesis are readily available. Thus, it is possible that in more limiting conditions, for example, under low nitrogen or carbon availability, the trends that we observed would be more pronounced. 

**Figure 5:**
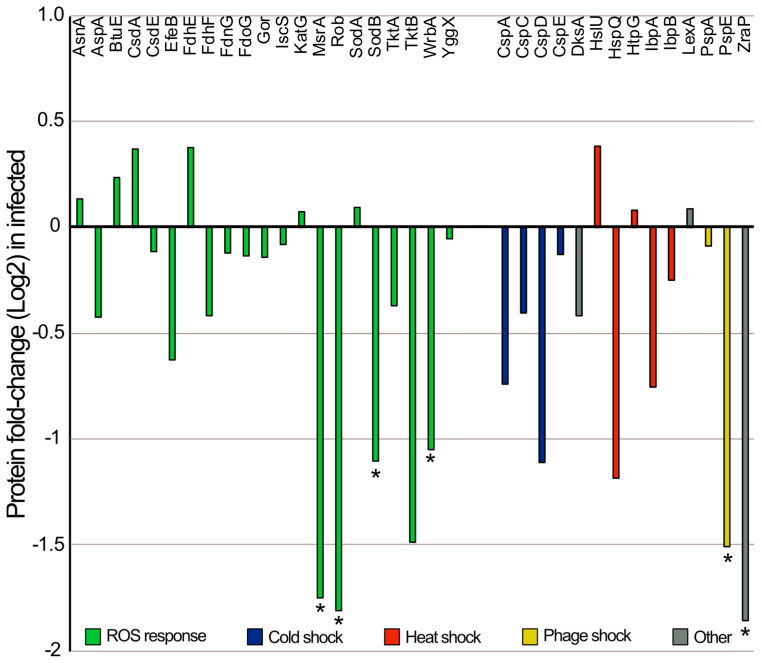
Fold change for the 33 detected proteins involved in the stress responses of *E. coli* cultures after 25 min of infection with phage EE09. Proteins whose abundance was significantly lower in the infected cultures are marked with asterisks.

Among the 14 proteins with a lower abundance in the infected samples compared to the non-infected controls, 5 were involved in genetic and information processes, 4 in metabolic processes, 4 in signaling processes, and 1 was a hypothetical protein (Figure 3b). In contrast to what observed for other coliphages that trigger the overexpression of *E. coli* oxidative stress response genes [17,66], four of the proteins with a significantly lower abundance in the infected cells compared to the controls (MsrA, SodB, WrbA, and Rob) are involved in protection against oxidative stress [67,68,69,70,71]. These conflicting results may be attributed to diverse factors or mechanisms. For example, different phages trigger different responses within the same host [20], and different responses are observed during the phage infection cycle [19]. Phage EE09 may have developed a defense mechanism to downregulate the reactive oxidative stress (ROS) response to manipulate the host’s defenses [72]. The latter hypothesis may also explain the significantly lower abundance of ZraP and PspE, which play key roles in the stress response and could have been downregulated during phage–host interactions at a certain stage of the infection cycle [73,74,75]. Based on this observation, we selected proteins present in both infected cultures and non-infected controls known to be involved in protection against reactive oxygen species and other stress-related proteins, such as temperature shock and phage shock proteins [66,76]. We detected 35 proteins in infected and control cultures that were involved in these processes (Figure 5). When we looked at the fold-change values, we observed that 74% of the proteins detected, involved in stress responses, were less abundant in the infected cultures compared to the controls (Figure 5). Moreover, six proteins were significantly less abundant in the infected cultures than in the controls. Hence, this suggests that EE09 infection downregulated the *E. coli* stress response. Potential advantages of decreasing the host stress response for the phage may be the reduction of the host defense mechanisms against phage infection and/or the reallocation of cellular resources towards the synthesis of new viral particles. 

## 4. Conclusions

In this study, we characterized phage vB_EcoS-EE09, of the genus *Dhillonvirus*, using cultivation, genomics, and proteomics. To the best of our knowledge, we hereby present the most comprehensive characterization of a phage within the *Dhillonvirus* genus to date. Moreover, we evaluated the differences in *E. coli* proteomes infected and not infected with phage EE09. We found significant differences in the abundance of 19 proteins, among which 5 involved in the response to reactive oxygen species and 3 involved in the general cellular stress response were significantly less abundant in the infected cultures compared to the non-infected controls. Additionally, we observed that proteins involved in the stress response tended to be less abundant in the infected cells compared to the controls, which suggests a survival mechanism of phage EE09. Even though the protocol that we developed here was used to compare proteins at a single time point under a specific growth condition, it may, in the future, be used to test further hypotheses and in more detailed, time-resolved studies. For instance, it could be used to test various conditions that could trigger the lysogenic cycle of temperate phages, or to assess how phage infection impacts diverse metabolic processes. Analyzing proteome datasets related to host–phage interactions to facilitate the specific detection of proteins is of great interest. This includes the identification of the potential overexpression of pathogenicity factors or host toxins, thereby allowing for the assessment of safety concerns, especially in the context of phage therapy and wastewater management. 

## Figures and Tables

**Table 1:** List of phage proteins detected via nMS/MS orbitrap analysis in cell-free phage lysates with their respective protein annotation and the peptides detected.

CDS vB_EcoS-EE09	Annotation	Protein Length (Amino Acids)	Protein Mass	Number of Detected Peptides	Coverage (%)
CDS8	Tail fiber protein	606	65.6 kDa	14	34
CDS10	Hypothetical protein	224	23.3 kDa	2	12
CDS12	Tail tip, host specificity protein J	1139	125.8 kDa	25	31
CDS13	Tail tip assembly protein	205	21.3 kDa	1	9
CDS14	Phage tail assembly protein	246	27.5 kDa	1	4
CDS15	Phage minor tail protein	262	28.8 kDa	8	44
CDS16	Tail protein	199	21.8 kDa	3	21
CDS17	Tail tape measure protein H	854	90.9 kDa	31	45
CDS21	Tape measure chaperone	103	11.0 kDa	2	19
CDS22	Major tail protein	241	25.6 kDa	3	16
CDS23	Hypothetical protein	140	15.0 kDa	2	18
CDS24	Putative tail protein	117	12.9 kDa	1	6
CDS27	Hypothetical protein	166	17.8 kDa	1	9
CDS28	Major capsid protein	366	38.4 kDa	8	28
CDS29	Capsid and scaffold protein	99	10.7 kDa	8	80
CDS30	Hypothetical protein	88	9.8 kDa	5	44
CDS34	Hypothetical protein	102	11.4 Kda	1	12
CDS35	Phage capsid and scaffold	367	41.0 Kda	3	11
CDS36	Minor tail protein	506	55.2 kDa	15	36
CDS37	Phage terminase	461	52.2 kDa	1	3
CDS39	Calcineurin-like phoshoesterase	380	42.8 kDa	3	11
CDS40	Hypothetical protein	137	13.6 kDa	3	28
CDS41	Putative phage lysin	163	18.2 kDa	5	41
CDS61	Hypothetical protein	766	30.7 kDa	2	11

## Data Availability

The mass spectrometric raw data and protein sequence databases were deposited in the PRIDE partner repository of the ProteomeXchange Consortium (http://proteomecentral.proteomexchange.org; accessed on 27 October 2023) [77] with the data set identifiers PXD046455 and 10.6019/PXD046455. The genome of Escherichia phage vB_EcoS-EE09 was deposited in the NCBI database under the accession number OR756193.

## Supplementary Materials

The following supporting information can be downloaded at https://www.mdpi.com/article/10.3390/microorganisms11112688/s1, Figure S1. Map and picture location of the isolation sampling site in the river Erpe, Berlin, right after the discharge of treated sewage water from WWTP Münchehofe. Image taken from Google Earth, earth.google.com/web/. Figure S2. Hierarchical viral clustering of EE09 for taxonomic classification using VirClust-based protein clustering (PC) and 1000 Bootstrap. For the clustering tree, phage genome sequences from the NCBI nt that resulted in a significant alignment with EE09 (E-value 0.0; >80% nucleotide identity; >75% query coverage) were selected and are shown (52 genomes). Figure S3. Protein-based phage similarity network of EE09 using vConTact 2.0 pipeline and the ProkaryoticViralRefSeq v94 virus database. Each circle represents different phages. Red represents EE09; orange indicates those phages that are close relatives of EE09 within the ViralRefSeq 94 database. Network visualization was generated using Cytoscape 3.9.1. Figure S4. Heatmap generated with VIRIDIC, showing the similarity values for phage EE09, highlighted in red, and its closest relatives. Closest relatives’ sequences were downloaded from the NCBI database, after a BLAST search. The results indicated that phage EE09 belongs to the same genus, Dhillonvirus (identity ~70%). Intergenomic similarity suggested that EE09 is a novel specie. Figure S5. Output results of the PhageTerm analysis of the genome of phage EE09 supporting the 5′ cos overhang. Figure S6. Results retrieved after BLAST alignment using BlastKoala for the proteins detected. We detected 1060 proteins in both EE09-infected *E. coli* cultures and the respective controls; 48 proteins were detected only in cultures infected with phage EE09, and 68 proteins were only detected in the control cultures. Figure S7. Relative abundances of the proteins detected via nLC-MS/MS Orbitrap in *E. coli* cultures 25 min post infection with phage EE09 (MOI 5) and their respective controls. Proteins were filtered as follows: master protein only, 1% FDR confidence in at least two replicates, >1 peptide. Proteins were classified based on BRITE functional hierarchies. (a) Relative abundances of the proteins that were detected in both control and infected cultures (bars labeled as “Control” and “Infected, respectively). (b). Relative abundances of the proteins detected only in the control (bar labeled as “Only Control”) or in the infected cultures (bar labeled as “Only Infected). Table S1. Genotype of the *Escherichia coli* strains used for the host range analysis of phage EE09. Table S2. The first five hits with phage EE9 after BLAST nt in the NCBI database according to the total score and E-value. Table S3. Read mapping statistics (short read) of the genome sequencing of Escherichia phage EE09. Table S4. Genome annotation of phage EE09.
