# Supplementary material for: Characterization of phage vB_EcoS-EE09 infecting E. coli DSM613 Isolated from Wastewater Treatment Plant Effluent and Comparative Proteomics of the Infected and Non-Infected Host
Source: Microorganisms. 2023 Nov 2;11(11):2688. doi: 10.3390/microorganisms11112688 (PMC10673088; doi:10.3390/microorganisms11112688)
Supplement: Supplementary file 1 [file microorganisms-11-02688-s001.zip › microorganisms-2673921-supplementary.pdf]

Supplementary information

Characterization of phage vB_EcoS-EE09 infecting *E. coli* DSM613 isolated from wastewater treatment plant effluent and comparative proteomics of the infected and non-infected host

Jimena Barrero-Canosa[1,*], Luyao Wang[1], Angelah Oyugi[1], Simon Klaes[2,3], Pascal Fischer[1], Lorenz Adrian[2,3], Ulrich Szewzyk[1] and Myriel Cooper[1]

1 Technische Universität Berlin, Institute of Environmental Technology, Chair of Environmental Microbiology, Straße des 17. Juni 135 Berlin, Germany
2 Technische Universität Berlin, Institute of Biotechnology, Chair of Geobiotechnology, Ackerstraße 76, 13355 Berlin, Germany
3 Helmholtz-Centre for Environmental Research GmbH - UFZ, Department of Environmental Biotechnology, Permoserstraße 15, 04318 Leipzig, Germany
***** Correspondence: Jimena.barrerocanosa@tu-berlin.de; +49 30 314 26827

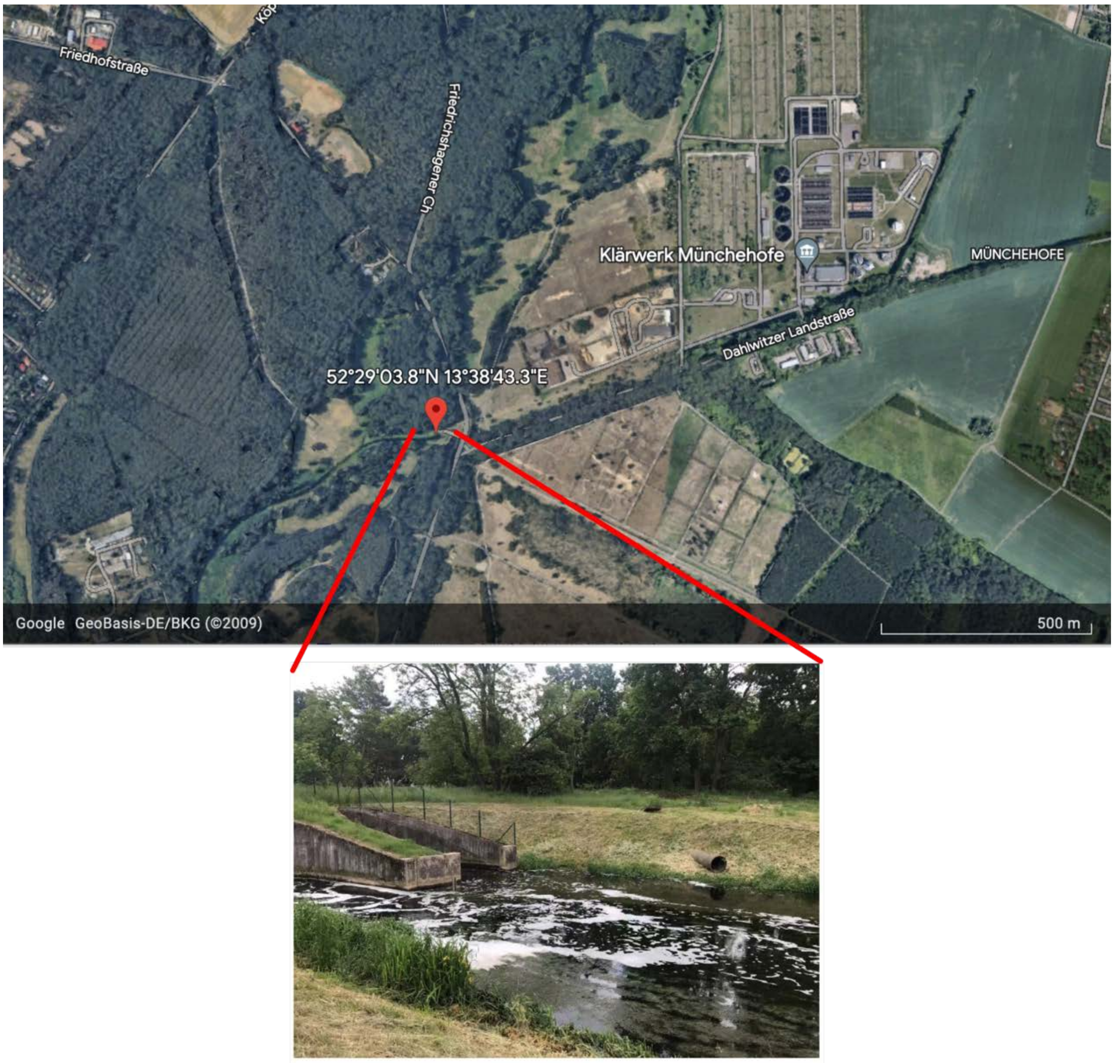

Figure S1. Map and picture location of the isolation sampling site in the river Erpe, Berlin, right after the discharge of treated sewage water from WWTP Münchehofe. Map image taken from *Google Earth August 2023*, earth.google.com/web/.

Table S1. Genotype of the *Escherichia coli* strains used for the host range analysis of phage EE09.

E. coli **strains**	**Genotype**	**Source**
BL 21	fhuA2 [lon] ompT gal (λ DE3) [dcm] ΔhsdS<br>λ DE3 = λ sBamHIo ΔEcoRI-B int:☹lacI::PlacUV5::T7 gene1) i21 Δnin5	CGSC Database
DH5 alpha	Δ(argF-lac)169, φ80dlacZ58(M15), ΔphoA8, glnX44(AS), deoR481, rfbC1, gyrA96(NalR), recA1, endA1, thiE1 and hsdR17.	CGSC Database
DSM 1103	Clinical Isolate	DSMZ Catalogue
DSM 5695	F+ met str, T1s T6s lambda-	DSMZ Catalogue
DSM 613	Parent Strain of several B derivatives	DSMZ Catalogue
DSM 4230	F- hsd R514 (rk-, mk-) sup F58, lac Y1 gal K2 gal T22 met B1 trp R55 l-	DSMZ Catalogue
HB101	F–, thi-1, hsdS20 (rB–, mB–), supE44, recA13, ara-14, leuB6, proA2, lacY1, galK2, rpsL20 (strr), xyl-5, mtl-1	CGSC Database

	(pRK2013,Km)	
HS	Δda– - (Commensal Isolate)	[1]
HS 996	F- mcrA Δ(mrr-hsdRMS-mcrBC) Φ80lacZ ΔM15 ΔLacX74 recA1 araD139 Δ(ara-leu)7697 galU galK rpsL (StrR) endA1 nupG fhuA::IS2	Thermo Fisher
JM 101	supE thi-1 Δ(lac-proAB) [F traD36 proAB lacIqZΔM15]	[2]
JM 109	endA1, recA1, gyrA96, thi, hsdR17 (rk–, mk+), relA1, supE44, Δ(lac-proAB), [F´ traD36, proAB, lacIqZΔM15]	Promega
K12	F+, lambda+	ATCC Collection
MG1655	F- lambda- *ilvG*- *rfb*-50 *rph*-1	UW E. coli Genome Project
	pcK218:jim4	
S17-1	TpR SmR recA, thi, pro, hsdR-M+RP4: 2-Tc:Mu: Km Tn7 λpir.	CGSC Database
SM10	thi thr leu tonA lacY supE recA::RP4-2-Tc::Mu Km λpir (pUX-BF13, Ap)	BCCM/GeneCorner
VH33	ΔptsH, ptsI, lacI, lacZ::loxP W3110 derivative that lacks PTS activity	[3]

Table S2. Top five hits when searching the NCBI nt database with the full sequence of phage EE9 as query using BLASTn.

Name	Total Score	Query Coverage	E value	Sequence similarity	Accession
Escherichia phage GeorgBuechner	62433	96%	0	91.90%	MZ501070.1
Escherichia phage SECphi18	62112	96%	0	94.23%	LT960609.1
Escherichia phage welsh	61872	94%	0	93.95%	MN850589.1
Escherichia phage Oekolampad	61816	96%	0	92.85%	MZ501095.1
Escherichia phage jat	61764	95%	0	93.93%	MN850650.1

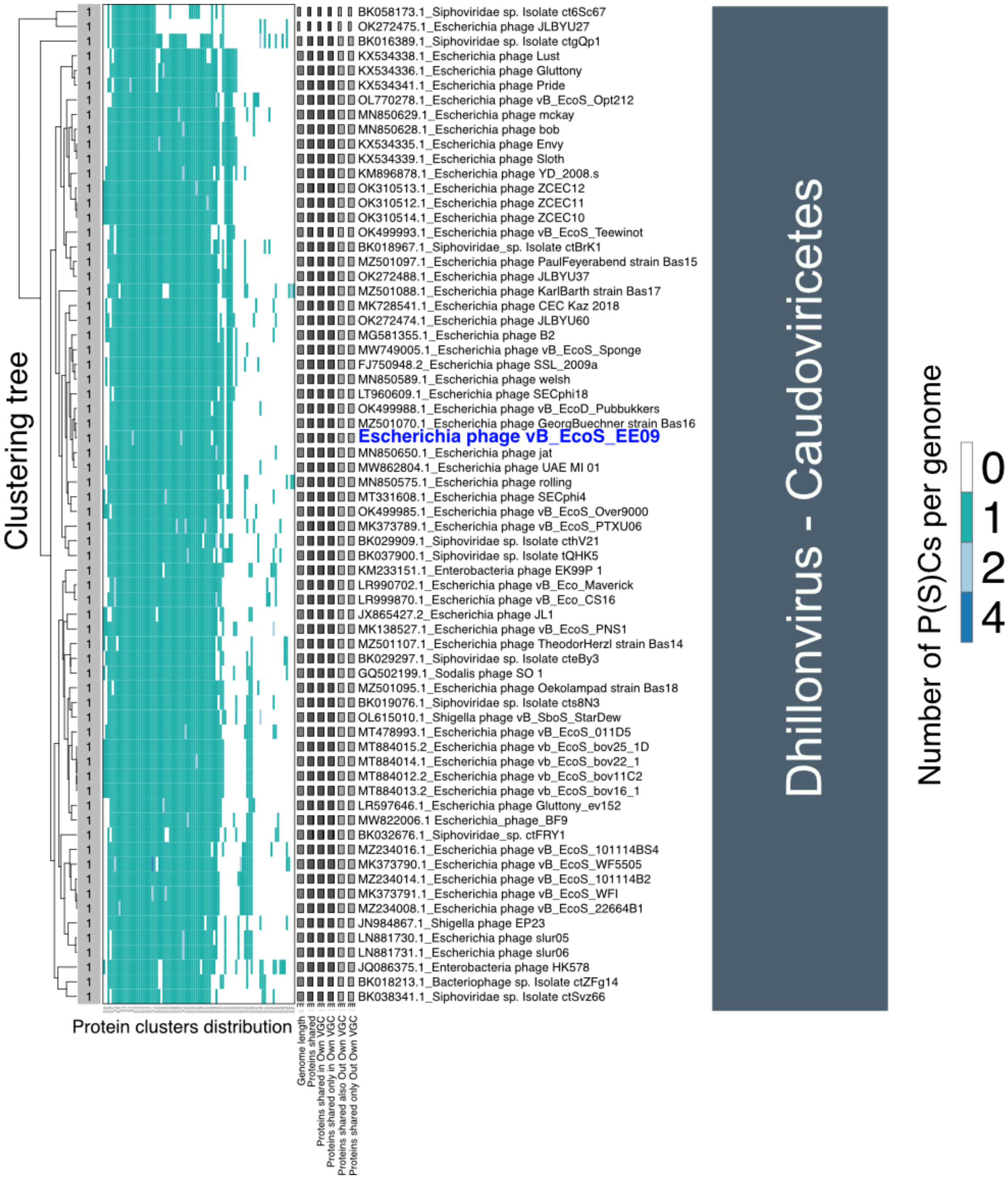

Figure S2. Hierarchical viral clustering of EE09 for taxonomic classification using VirClust-based protein clustering (PC) and 1000 bootstrap replications [4]. For the clustering tree, phage genome sequences from the NCBI nt that resulted in a significant alignment with EE09 (E-value 0.0; >80% nucleotide identity; >75% query coverage) were selected and are shown (52 genomes).

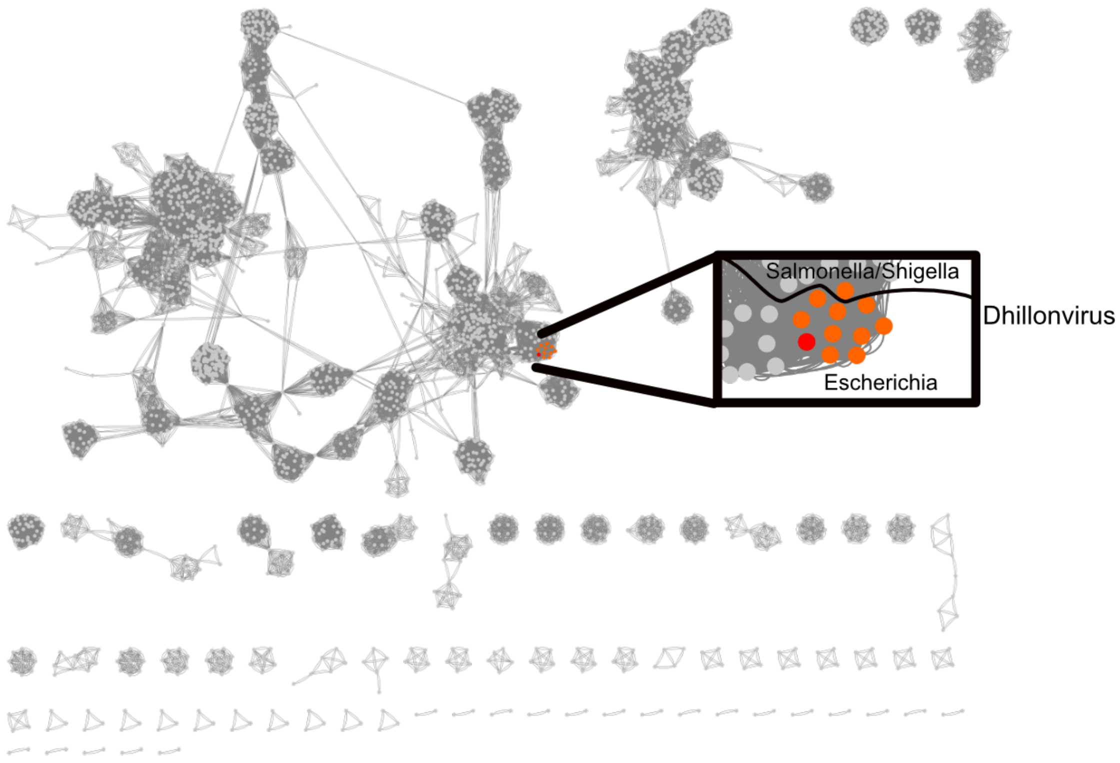

Figure S3. Protein-based phage similarity network of EE09 using vConTact 2.0 pipeline and the ProkaryoticViralRefSeq v94 virus database. Each note (circle) represents different phages. Red node represents EE09; orange indicates those phages that are close relatives of EE09 within the ViralRefSeq v94 database. Network visualization was generated using Cytoscape 3.9.1.

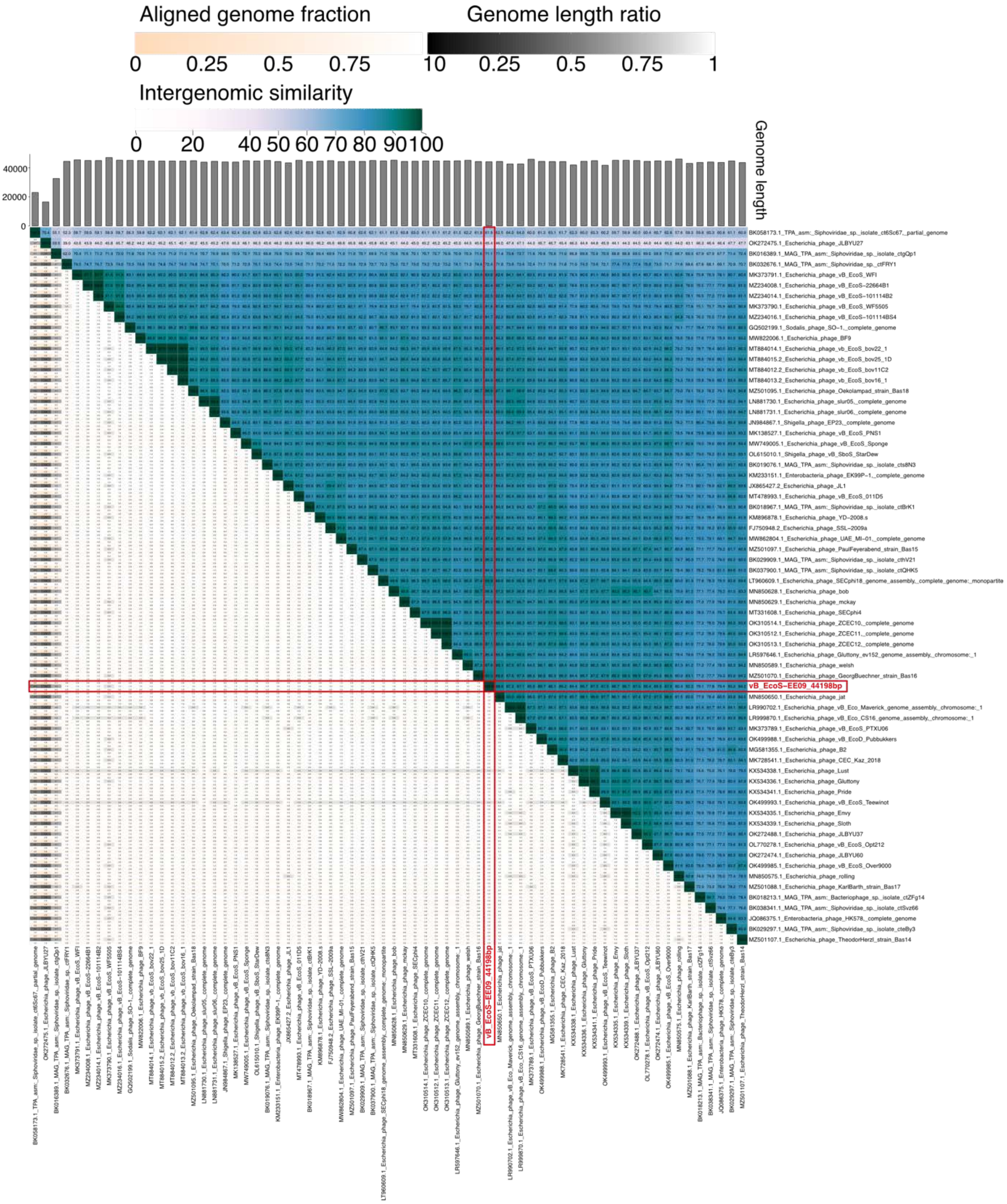

Figure S4: Heatmap generated with VIRIDIC[5], showing the similarity values for phage EE09, highlighted in red, and its closest relatives. Closest relatives' sequences were downloaded from the NCBI database, after a BLAST search. The results indicated that phage EE09 belongs to the same genus (identity ~70%). Intergenomic similarity suggested that EE09 is a novel species.

Table S3. Read mapping statistics (short read) of the genome sequencing of Escherichia phage EE09.

Read mapping statistics	Total reads	Mapped reads	Average coverage	Insert size median
EE09 phage genome	3692516	3571163	11681.26	435

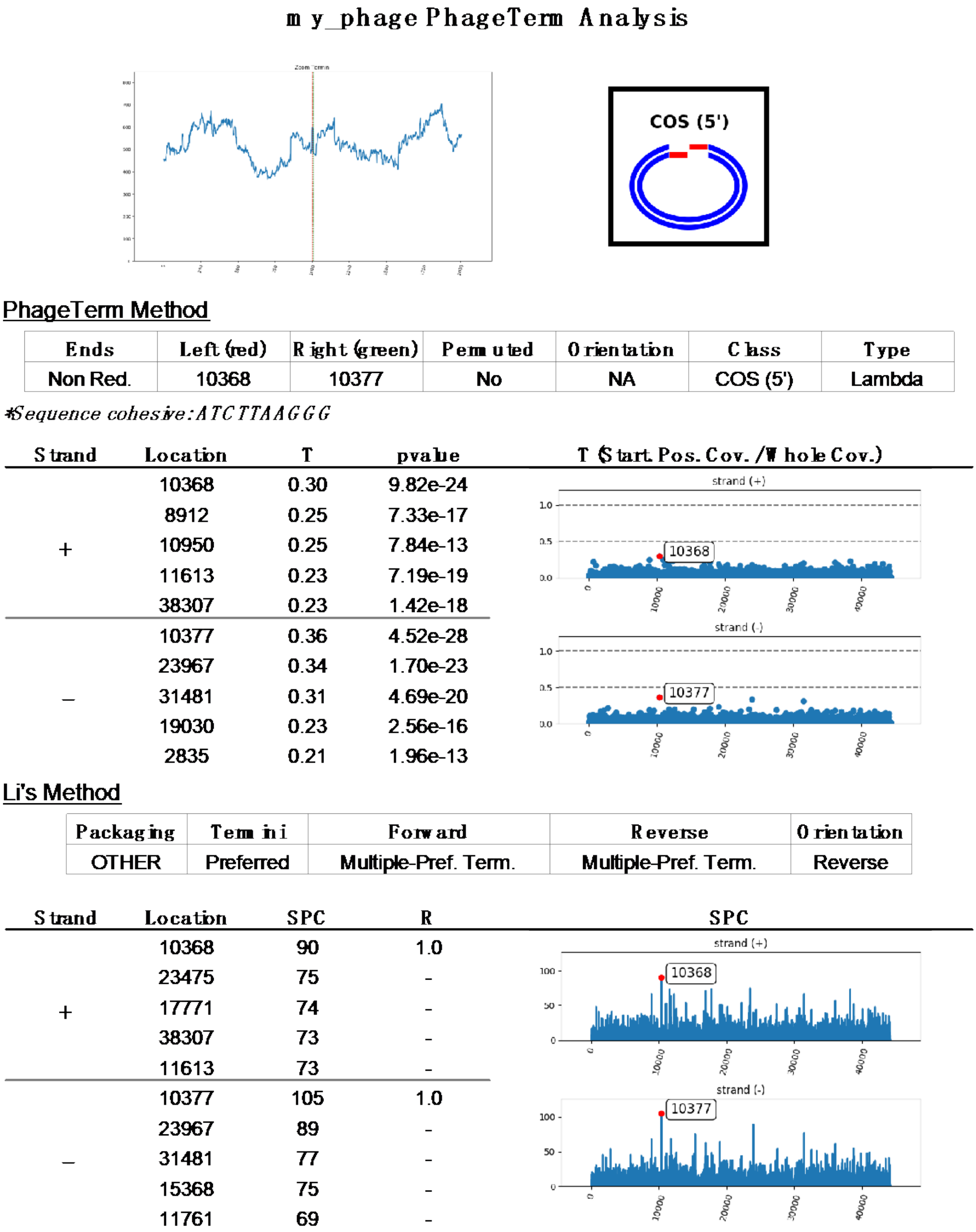

Figure S5. Output results of the PhageTerm analysis [6] of the genome of phage EE09 supporting the 5’ cos overhang.

Table S4 Genome annotation of phage EE09.

ORF		Starting position	Ending position	bp	Direction	Predicted function
1	CDS	53	295	243	=>	Hypothetical protein
2	CDS	296	760	465	=>	Hypothetical protein
3	CDS	747	1232	486	=>	Phage-associated DNA N-6-adenine methyltransferase
4	CDS	1213	1437	225	=>	Hypothetical protein
5	CDS	1434	1904	471	=>	Polynucleotide kinase/phosphatase
6	CDS	1966	2487	522	=>	Hypothetical protein
7	CDS	2530	3105	576	=>	Hypothetical protein
8	CDS	3132	4952	1821	<=	Putative tail fiber protein
9	CDS	5033	5251	219	=>	Putative phage super-infection exclusion protein
10	CDS	5271	5945	675	<=	Hypothetical protein
11	CDS	5945	6247	303	<=	Hypothetical protein
12	CDS	6284	9703	3420	<=	Phage tail tip, host specificity protein J
13	CDS	9700	10.317	618	<=	Phage tail tip, assembly protein I
14	CDS	10.308	11.048	741	<=	Phage tail assembly protein
15	CDS	11.051	11.839	789	<=	Phage minor tail protein
16	CDS	11.836	12.435	600	<=	Putative tail protein
17	CDS	12.472	15.036	2565	<=	Phage tail, tail length tape-measure protein H
18	CDS	15.110	15.421	312	<=	Hypothetical protein
19	CDS	15.421	15.537	117	<=	Hypothetical protein
20	CDS	15.518	15.673	156	<=	Hypothetical protein
21	CDS	15.735	16.097	363	<=	Tape measure chaperone
22	CDS	16.168	16.893	726	<=	Phage major tail protein
23	CDS	16.955	17.377	423	<=	Hypothetical protein
24	CDS	17.377	17.970	594	<=	Putative tail protein
25	CDS	17.972	18.325	354	<=	Head-tail joining protein
26	CDS	18.312	18.419	108	<=	Hypothetical protein
27	CDS	18.421	18.921	501	<=	Hypothetical protein
28	CDS	18.980	20.080	1101	<=	Phage major capsid protein of Caudovirales
29	CDS	20.178	20.477	300	<=	Capsid and scaffold protein
30	CDS	20.450	20.716	267	<=	Hypothetical protein
31	CDS	20.761	20.883	123	<=	Hypothetical protein
32	CDS	20.948	21.628	681	=>	Hypothetical protein
33	CDS	21.643	21.973	331	<=	Hypothetical protein
34	CDS	21.776	22.084	309	<=	Hypothetical protein
35	CDS	22.077	23.180	1104	<=	Phage capsid and scaffold
36	CDS	23.164	24.684	1521	<=	Putative minor tail protein
37	CDS	24.696	26.081	1386	<=	Phage terminase
38	CDS	26.081	26.653	573	<=	Phage terminase, small subunit
39	CDS	26.763	27.905	1143	<=	Putative Calcineurin-like phosphodiesterase
40	CDS	27.998	28.411	414	<=	Hypothetical protein
41	CDS	28.428	28.919	492	<=	Putative phage lysin
42	CDS	28.906	29.151	246	<=	Putative holin-like protein
43	CDS	29.148	29.438	291	<=	Putative holin-like class II protein
44	CDS	29.477	29.764	288	<=	Hypothetical protein

45	CDS	29.761	29.901	141	<=	Hypothetical protein
46	CDS	29.984	30.556	573	<=	Hypothetical protein
47	CDS	30.553	30.705	153	<=	Hypothetical protein
48	CDS	30.702	30.971	270	<=	Hypothetical protein
49	CDS	30.981	31.460	480	<=	Hypothetical protein
50	CDS	31.429	31.593	165	<=	Hypothetical protein
51	CDS	31.713	31.883	171	<=	Hypothetical protein
52	CDS	31.941	33.806	1866	<=	Putative helicase primase
53	CDS	33.803	34.282	480	<=	Phage replicative DNA helicase
54	CDS	34.293	34.622	330	<=	Putative transposase
55	CDS	34.644	34.754	111	=>	Hypothetical protein
56	CDS	34.845	35.543	699	=>	Hypothetical protein
57	CDS	35.592	35.843	252	=>	Hypothetical protein
58	CDS	35.844	36.032	189	=>	Hypothetical protein
59	CDS	36.029	37.462	1434	=>	Putative nuclease superfamily protein
60	CDS	37.455	37.697	243	=>	Hypothetical protein
61	CDS	37.773	38.573	801	=>	Hypothetical protein
62	CDS	38.632	38.802	171	=>	Hypothetical protein
63	CDS	38.799	40.958	2160	=>	DNA polymerase I
64	CDS	40.991	41.236	246	=>	Hypothetical nuclease
65	CDS	41.277	41.450	174	=>	HNH endonuclease
66	CDS	41.489	41.713	225	=>	Hypothetical protein
67	CDS	41.718	43.142	1425	=>	Putative phage DNA helicase
68	CDS	43.139	43.849	711	=>	Hypothetical protein
69	CDS	43.846	44.169	324	=>	Putative HNH endonuclease

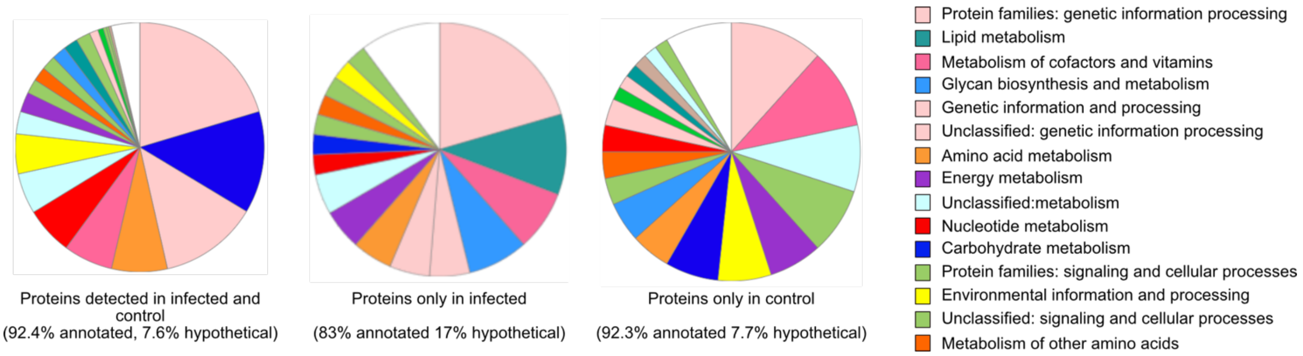

Figure S6. Results were retrieved after BLAST alignment using BlastKoala for the detected proteins. We detected 1060 proteins in both EE09-infected *E. coli* cultures and their respective non-infected controls; 48 proteins were detected only in cultures infected with phage EE09, and 68 proteins were only detected in the non-infected control cultures.

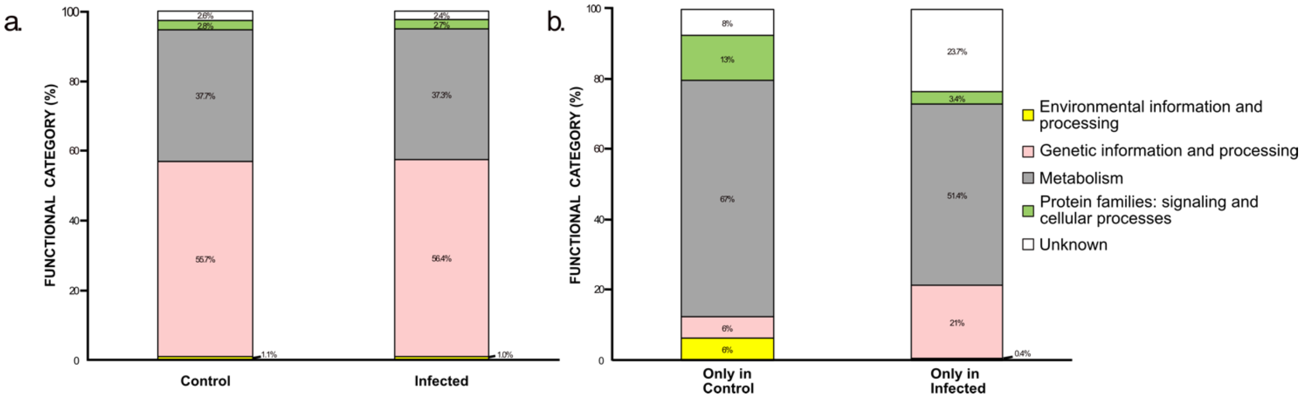

Figure S7. Relative abundances of the proteins of *E. coli* cultures detected via nLC-MS/MS Orbitrap in *E. coli* cultures 25 minutes post infection with phage EE09 (MOI 5) and their respective controls. Proteins were filtered as follows: master proteins only, 1% FDR confidence in at least two replicates, >1 peptide. Proteins were classified based on BRITE functional hierarchies. (a) Relative abundances of the proteins that were detected in both control and infected cultures (bars are labeled as “Control” and “Infected”, respectively). (b) Relative abundances of the proteins detected only either in the control (bar labeled as “Only Control”) or in the infected cultures (bar labeled as“Only Infected).

Supplementary text

PROTOCOL FOR PROTEIN ANALYSIS OF CELLS INFECTED WITH BACTERIOPHAGES

The protocol was optimized based on [7-10]. In the following, the steps for phage infection, sample collection, and cell disruption are described.

Here, the protocol is described with the media and incubation temperatures and times used for our protein analysis with *E. coli* and Escherichia phage vB_EcoS-EE09. However, the selection of media and incubation conditions should be adapted to the bacterial strain to be studied. We suggest that the one-step growth curve and proteomics sampling are done simultaneously using the same culture.

MATERIALS

- **Working buffer and solutions:**

1. **100 mM Ammonium bicarbonate Ambic buffer, pH 7.9 (Ammonium hydrogen carbonate M = 79.06 g mol^{-1}) Buffer:** dissolve 0.79 g Ambic in 100 ml Milli Q water and sterile-filtered using 0.2 µm pore-size filter. pH should be 7.9. The pH of the buffer will rise slowly. A fresh buffer should be prepared weekly and stored at 4 °C.
2. **Native lysis Buffer:** Prepare native lysis buffer by mixing 20 mM HEPES (AppliChem), 150 mM potassium chloride (MERCK), and 10 mM magnesium chloride (MERCK) at pH 7.5. The buffer should be freshly prepared.
3. **Sodium deoxycholate/deoxycholic acid sodium salt, 97% (DOC), (Sigma-Aldrich):** DOC solution is 10% (w/vol) DOC in LC/MS-grade water. Vortex the solution vigorously to dissolve the DOC.
4. **Dithiothreitol (DTT; M = 154.25 g mol$^{-1,}$ PanReac AppliChem):** Prepare 700 mM DTT solution by dissolving DTT in LC-MS-grade water. Prepare fresh before use.
5. **2-iodoacetamide (IAA; M = 184.96 g mol^{-1}) (MERCK):** Prepare 700 mM IAA solution by dissolving IAA in LC-MS-grade water. Prepare fresh before use.
6. **Trypsin suspension buffer:** 50 mM acetic acid (Promega Sequencing Grade) for the reconstitution of the lyophilized trypsin.
7. **0.1 µg µl^{-1} trypsin stock solution:** trypsin (Promega sequencing grade) is delivered as aliquots of 20 µg powder in glass vials. To be dissolved in 200 µl of 50 mM acetic acid (“Trypsin suspension buffer”).
8. **Formic acid (Honeywell):** Pure – 100%, LC-MS grade.
9. **LC/MS-grade water (PanReac AppliChem) or (ddH_2O Arium® proVF Sartorius)**
10. **10% (v/v) formic acid prepared using LC-MS grade water**
11. **Acetonitrile LC-MS- grade (Honeywell)**
12. **Bovine Serum Albumin (BSA) Internal Standard, 2.0 mg ml^{-1} in 0.9% aqueous NaCl solution containing sodium azide. (Thermo Scientific)**
13. BSA working solution: Dilute 2 µl BSA standard (2.0 mg ml^{-1}) in 98 µl of 50 mM Ambic buffer to a final concentration of 40 µg ml^{-1}

14. **Saline-Magnesium Buffer (SMG Buffer) plus Gelatine:** Composition: 50 mM Tris-HCl (pH 7.5) containing 100 mM NaCl (Carl ROTH), 8.1 mM $MgSO_4$ (MERCK), and 0.01% (w/v) gelatine): For 1 l dH_2O: dissolve 5.8 g NaCl, 2 g $MgSO_4$* 7 H_2O, 50 ml of 1 M Tris-HCl (pH 7.5), and 5 ml of 2% (w/v) gelatine. Autoclave at 121 °C for 15 min (103 kPa).

- **Culture media:**

LB is prepared as follows:

For 1 l dH_2O: dissolve 10 g of peptone (Carl ROTH), 5 g yeast extract (Sigma-Aldrich), and 10 g NaCl (Carl ROTH), mix well, adjust pH to 7 by titration using 1 N sodium hydroxide. Autoclave at 121 °C for 15 min (103 kPa). LB plates and top agar are produced by adding 15 g and 7 g of Agar (Carl ROTH) respectively to the media before autoclaving.

- **Glassware and plastic ware:**

1. Two Erlenmeyer flasks of 100 ml.
2. Erlenmeyer flasks of 50 ml.
3. Pipette tips of different volumes 5 ml, 1000 µl, 100 µl, 10 µl.
4. 5 µl fixed volume pipette with a glass tip.
5. 1 µl fixed volume pipette with a glass tip.
6. LB plates and soft agar.
7. Sterile measure cylinders of 25 ml.
8. 1.5 ml spectrophotometer cuvettes.
9. Sterile pipette tips.
10. 2 ml sterile centrifuge tubes.
11. 1.5 ml sterile centrifuge tubes.
12. Universal pH indicator paper.
13. 100 µl Pierce C_{18} tips (Thermo Fisher Scientific, Catalogue number: 87784).

- **Laboratory equipment:**

1. Incubators at 37 °C.
2. Shakers at 110 RPM.
3. Water baths: at different temperatures at 37 °C, 55 °C and 80 °C.
4. Ice bath.
5. Tabletop centrifuge.
6. Vacuum centrifuge.
7. Mini centrifuge.
8. Ultrasonic bath.
9. Spectrophotometer.
10. µVolume Spectrophotmeter DS-11 (DeNovix)
11. Pipettes of 5 ml, 1000, 100 and 10 µl volumes.
12. Liquid nitrogen and a cryogenic container that can be placed close to the sterile bench during the experiment.
13. UltiMate 3000 RSLCnano high-performance nano-UPLC system (Thermo Scientific) equipped with an Acclaim PepMap 100 C_{18} trap column (75 µm x 2 cm, 3 µm material, Thermo Scientific) and an Acclaim PepMap 100 C_{18} analytical column (75 µm x 25 cm, 3 µm material, Thermo Scientific)

14. Orbitrap Fusion Tribrid mass spectrometer (Thermo Scientific) equipped with a TriVersa NanoMate nano-electrospray ionization (nano-ESI) ion source (Advion)
15. Proteome Discoverer v2.4 (Thermo Fisher Scientific), using the Sequest HT database search engine.
16. Excel Microsoft
17. https://huygens.science.uva.nl/VolcaNoseR2/ [11].

PRIOR TO PROTEOMIC EXPERIMENT

- **<u>Preparation of precultures</u>**

1. Streak the bacterium from the glycerol stock of the strain collection on two LB-agar plates.
2. Incubate overnight at 37 °C, store for a maximum of 4 weeks at 4 °C.
3. Pick a single colony from a plate using a sterile microbial loop and inoculate 20 ml of LB-media in a 50 ml-Erlenmeyer flask. Incubate overnight at 37 °C, 110 rpm.
4. Inoculate a new 50 ml-Erlenmeyer flask with 20 ml of fresh LB with 20 µl of the overnight culture from step 3. Incubate overnight at 37 °C, 110 rpm.
5. Repeat step 4 to permit the cells to adapt to the medium and allow growth synchronization.

- **<u>Quantification of phage lysates</u>**

1. Phage lysates are assayed by the double agar overlay method described by [12]. Prepare 1:10 dilutions of the phage lysate by adding 0.1 ml of the lysate into 0.9 ml SMG buffer. Prepare dilutions from 10^{-1} to 10^{-7}. The dilutions that will need to be prepared will depend on the estimated (expected) titer of the phage lysate, from our experience, to obtain 30 to 300 plaques generally dilutions 10^{-4} to 10^{-7} should be used. Notice that this may vary for other phages.
2. On a tube with molten soft agar, add 0.1 ml of the dilution and 0.1 ml of a log-phase culture of *E. coli* (OD approx. 0.2), mix by gently swirling.
3. Pour the soft agar on top of an LB plate, and allow the soft agar to solidify. Prepare three plates per dilution.
4. Invert solidified plates and incubate for a minimum of 18 h (overnight) at 37 °C and subsequently plaque-forming units ml^{-1} (PFU ml^{-1}) are counted.
5. Count the lytic plaques on the plates that were prepared with the right dilution, that is, the plates in which 30 to 300 plaques are observed. Calculate the average number of plaques for the triplicates. Once the average of counted plaques is determined, the phage titer is calculated using the following formula:

$$\text{PFU ml}^{-1} = \frac{Average\ number\ \ of\ plaques \times\ Dilution\ factor}{Volume\ used\ [ml]}$$

- **<u>Calibration curves of OD vs CFU ml^{-1}</u>**

Colony Forming Unit (CFU) Assay was used for the determination of the host cells' growth curves as described in [**Error! Reference source not found.**].

1. Grow bacterium in LB or preferred broth and sub-culture at least twice before the CFU ml^{-1} assay is carried out.

2. After the second sub-culturing, inoculate 200 ml of fresh sterile media with 200 µl of the overnight culture.
3. Collect 1 ml of the sample every hour and measure the optical density at 600 nm using a spectrophotometer. Use sterile broth to calibrate the spectrophotometer.
4. Simultaneously, dilute the batch culture through serial dilutions of 1:10 by transferring 0.1 ml of the culture into subsequent tubes containing 0.9 ml of 0.85% (w/v) NaCl.
5. Pipette and spread 0.1 ml of the dilutions onto LB agar plates, prepare triplicates per dilution.
6. Invert plates and incubate for a minimum of 18 h at 37 °C.
7. Count the colonies on plates prepared with the right dilution, that is, the plates in which 30 to 300 plaques are observed. Calculate the average number of colonies for the triplicates.
8. The CFU ml^{-1} is determined using the following formula:

$$\text{CFU ml}^{-1} = \frac{Average\ number\ of\ colonies \times Dilution\ Factor}{Volume\ \ [ml]}$$

9. Prepare three biological replicates per culture by repeating steps 1 to 8.
10. Produce a standard curve by plotting the OD against the average (from biological triplicates) CFU ml^{-1}. Generate a linear regression, which can then be used to relate these two units so that the optical density can readily be used to determine the bacterial cell count in the culture.

METHODS

- **Preparation of batch cultures for phage infection**

1. For the batch culture, inoculate 70 µl of the (twice sub-cultured) pre-culture in 70 ml of pre-warmed (in a 37 °C water bath) LB media in a 100 ml Erlenmeyer flask. Incubate at 37 °C, orbital shaking at 110 rpm.
2. Track the optical density (OD) regularly, approx. every 20 min, with a spectrophotometer at wavelength 600 nm until an OD of ~ 0.2 is reached.
3. Once an OD of ~0.2 is reached, divide the culture equally (25 ml per culture) using a sterile volumetric cylinder into 2 sterile 50 ml Erlenmeyer flasks labeled “control” and “infected”.
4. Convert the OD to CFU ml^{-1} using a calibration curve that should have been empirically determined previously (see protocol Calibration curves of OD vs CFU ml^{-1}).
5. Calculate the volume of the lysate needed to infect the bacterium with the desired multiplicity of infection, MOI, using the next equation:

$$Volume\ phage\ lysate\ (ml) = \frac{MOI\ x\ \frac{CFU^*}{ml}\ x\ \ Volume\ culture\ (ml)}{Phage\ titer\ (\frac{PFU}{ml})}$$

   *CFU ml^{-1} calculated after OD.
6. For the “infected” flask add the volume calculated in the previous step of the phage lysate in SMG buffer to infect the culture with the desired MOI. For the “control”

flask, add the same volume of sterile SMG buffer. Record the infection time and start a timer to track the infection cycle.

7. Incubate “infected” and “control” cultures at 37 °C shaking at 110 rpm.

- **Sampling for proteomic analysis**

1. After 25 min of infection, mix briefly and collect 2 ml of the control and infected cultures into separate sterile 2 ml reaction vials in triplicates.
2. Centrifuge at 21 910 g for 2 min and discard the supernatant.
3. Wash the pellet with 1 ml 100 mM Ambic buffer, centrifuge at 21 910 g for 1 min, and discard the supernatant.
4. Repeat the washing and centrifugation step and discard the supernatant.
5. Flash freeze the pellet in liquid nitrogen and store at -80 °C until processing.

- **Disruption of bacterial cells for protein extraction:**

1. Resuspend the flash frozen pellets in 20–50 µl of native lysis buffer depending on the pellet size. (Samples should be placed in an ice bath).
2. Run five freeze/thaw cycles. Use liquid nitrogen for the freezing and thaw in a thermal shaker at 40 °C for 2 min and 750 rpm. Make sure the suspension is completely defrosted during each cycle.
3. Place in an ultrasonic bath for 30 sec.
4. Centrifuge the sample twice for 10 min at 16 000 g at 4 °C to remove cell debris. Transfer the supernatant to a new tube.
5. Estimate the protein concentration using a DS-11 µVolume spectrophotometer (DeNovix) at 280 nm using native lysis buffer as blank (Note: for complex samples, estimated protein can vary depending on the method. Protein concentrations given in this protocol are always based on measuring absorption at 280 nm with 1 A = 1 mg ml^{-1}).
6. Dilute the protein extract to 1 mg ml^{-1} with native lysis buffer.
7. Add a peptide or protein internal standard the user is familiar with, for which differential proteolytic patterns are known and ideally which does not occur naturally in the biological extract. (2 µl of BSA working solution, resulting in an end concentration of 80 ng of Bovine Serum Albumin (BSA) was added into our samples as internal standard).
8. Add DOC to a final concentration of 5% (w/vol), using 10% (w/vol) stock DOC solution. You might observe the sample becoming a whitish gel.

- **Phage Lysate Protein Extraction**

1. Centrifuge 25 ml of phage lysate with a high titer, ideally 10^{10} PFU ml^{-1}, at 87 000 g at 4 °C for 2 h.
2. Discard the supernatant and resuspend the phage particles in 100 µl native lysis buffer and incubate overnight at 4 °C.
3. Run five freeze/thaw cycles. Use liquid nitrogen for the freezing and thaw in a thermomixer at 40 °C for 2 min and 750 rpm. Make sure the suspension is completely defrosted during each cycle.
4. Place in an ultrasonic bath for 30 s (short spin afterwards).

5. Centrifuge the sample for 10 min at 16 000 g at 4 °C to remove cell debris. Transfer the supernatant to a new tube.
6. Estimate the protein concentration using a DS-11 µVolume spectrophotometer (DeNovix) at 280 nm using the native lysis buffer as blank. (Note: For complex samples, estimated protein concentrations can vary depending on the method. Protein concentrations given in this protocol are always based on measuring absorbance at 280 nm with 1 A = 1 mg ml^{-1})
7. Add a peptide or protein internal standard the user is familiar with, for which differential proteolytic patterns are known and ideally which does not occur naturally in the biological extract. (2 µl of BSA working solution, resulting in 80 ng of Bovine Serum Albumin (BSA) was added into our samples as internal standard).
8. Add DOC to a final concentration of 5% (w/vol), using 10% (w/vol) stock DOC solution. You might observe the sample becoming a whitish gel.

- **<u>Trypsin Digestion</u>**

1. *Reduction of cysteine-SH groups:* Reduce the cysteine residues by adding DTT to a final concentration of 12 mM, then incubate the tubes for 30 min at 37 °C with slight agitation on a thermomixer at 400 rpm.

2. *Cysteine acetamidylation:* Alkylate the reduced cysteine residues by adding IAA to a final concentration of 40 mM. Incubate the tubes for 45 min at 20 °C in the dark with slight agitation on a thermomixer at 400 rpm. Note, that IAA is light-sensitive.

3. Dilute the sample to 1% (w/vol) DOC by adding 4 volumes of 100 mM Ambic buffer.

4. *Trypsin digestion:* Work with the trypsin in an ice bucket to prevent auto-proteolysis. Add 6.3 µl of trypsin stock solution resulting in 0.63 µg trypsin. Incubate the tubes at 37 °C overnight under agitation at 800 rpm.

5. Stop the digestion and precipitate the DOC by adding 100% (vol/vol) formic acid to a final concentration of 2.5% (vol/vol). Verify that the pH of the sample is <3.

6. DOC will form a white precipitate upon acidification. To remove the precipitate, centrifuge the sample at 16 000 g for 10 min at room temperature. Carefully transfer the supernatant and repeat the centrifugation step until there is no precipitate left in the sample as DOC may interfere with MS measurement.

  - **<u>Preparation for LC-MS/MS Analysis:</u>**

- Peptide Cleanup

1. Use the 100 µl Pierce C_{18} tip to desalt the samples. Load, wash, and elute the peptide mixtures, as recommended by the manufacturer with slight

modifications. (https://assets.thermofisher.com/TFS-Assets/LSG/manuals/MAN0011713_Pierce_C18_Tip_UG.pdf)

2. The wetting and equilibrating solutions are prepared as follows:
    1. 100% acetonitrile LC/MS-grade
    2. 80% acetonitrile in 0.1% formic acid in LC/MS-grade water
    3. 50% acetonitrile in 0.1% formic acid in LC/MS-grade water
    4. 30% acetonitrile in 0.1% formic acid in LC/MS-grade water
    5. 0.1% formic acid in LC/MS-grade water as the equilibrating solution
    6. 5% acetonitrile in 0.1% formic acid in LC/MS-grade water as the rinse solution
    7. 30% elution buffer (30% acetonitrile, 1% formic acid in LC/MS-grade water)
    8. 60% elution buffer (60% acetonitrile, 1% formic acid in LC/MS-grade water)
3. Desalting and elution of the peptide samples are done as follows:
    1. Wet the C_{18} tip by aspirating and discarding 200 µl of 100% acetonitrile solution. Do this step two times.
    2. Repeat step (1) for solutions 80%, 50%, and 30% acetonitrile in 0.1% formic acid solutions.
    3. Equilibrate by aspirating and discarding 200 µl of 0.1% formic acid. Do this step two times.
    4. Bind the peptide sample to the C_{18} material of the tip:
        - Pipette 200 µl of the sample into an intermediate glass vial, pipette up and down five times in the intermediate vial and discard this 200 µl sample. Repeat this step until all the sample in the sample tube is processed.
    5. Rinse the C_{18} tip by aspirating and discarding 200 µl of the rinse solution. Do this step two times.
    6. Elute the peptide by first pipetting up and down all liquid in the 30% elution solution five times and then five times in the 60% elution solution. At the end of the fifth pipetting in the 60% solution, pipet all liquid and transfer into the 30% elution tube.

4. Evaporate the solvent from the eluted peptides using a vacuum centrifuge at 1000 rpm, 20 mbar for a minimum of 1.5 h.
5. Store the dried peptides at -20 °C if not directly injected into the LC-MS/MS.

- Peptide resuspension

1. Resuspend the peptides in 0.1% (v/v) formic acid to a final concentration of ~ 4 mg ml^{-1}. Determine the volume of 0.1% formic acid to add based on the protein concentration estimated via absorbance at 280 nm after cell disruption.
2. Sonicate the peptide solution for 30 s and then centrifuge for 10 min at 16 000 g at room temperature.
3. Transfer the supernatant to an MS vial for subsequent MS analysis.

- **<u>Data Acquisition and Analysis</u>**

1. Inject 3 µl of the desalted peptide solution and separate via an UltiMate 3000 RSLCnano high-performance nano-UPLC system (Thermo Scientific) coupled to an Orbitrap Fusion Tribrid mass spectrometry (Thermo Scientific) via a TriVersa NanoMate nano-electrospray ionization (nano-ESI) ion source (Advion).
2. Load the peptides onto the Acclaim PepMap 100 C_{18} trap column (75 µm x 2 cm, 3 µm material, Thermo Scientific) with of 3.2% (v/v) acetonitrile in water and 0.1% formic acid at a flow rate of 5 µl min^{-1} for three min.
3. Switch the trap column into line with the Acclaim PepMap 100 C_{18} analytical column (75 µm x 25 cm, 3 µm material, Thermo Scientific) heated up to 35 °C.
4. Separate the peptides at a flow rate of 0.3 µl min^{-1} with a gradient of 145 min starting with 3.2% and increasing to 72% (v/v) acetonitrile in water and 0.1% (v/v) formic acid. Set the ion source in positive mode, and the spray voltage to 2.4 kV. Set the source temperature at 275 °C.
5. Run the mass spectrometer with a cycle time of 3 sec and in a data-dependent mode. Internal mass calibration is done with a lock mass of 445.12003 *m/z*. Precursor ions were scanned over a range of 350–2000 *m/z* with a resolution of 120 000, automatic gain control (AGC) target of 4 x 10^5 ions, and a maximum injection time of 50 ms in the Orbitrap mass analyzer. Select and isolate precursor ions with a minimum intensity of 5 x 10^4 and a charge state of +2 and +3. They are isolated by the quadrupole in a window of 1.6 *m/z* accumulating to an AGC target of 5 x 10^4 ions with a maximum injection time of 54 ms. Isolated precursor ions are fragmented using higher energy collisional dissociation (HCD) at 30% relative collision energy. Fragment ions are scanned with the Orbitrap analyzer at a resolution of 30 000. Precursor ions with the same mass (± 10 ppm) are excluded from further precursor selection for 30 s [10].
6. Analyze mass spectrometric raw data in Proteome Discoverer v2.4 (Thermo Fisher Scientific). A database search of the spectra was performed using Sequest HT with the protein databases for *E. coli* DSM613 protein database retrieved from the ATCC Genome portal (https://genomes.atcc.org/; culture col. no.: ATCC 11303 in the American Type Culture Collection, equivalent to DSM 613 of the German collection*)*, common laboratory contaminants, and the protein databases for phage EE09. Using the following settings:
    a. Mass tolerances of ±3 ppm and ±0.1 Da for precursor and fragment ions, respectively.
    b. Search restricted to peptides with a length of 6–144 amino acids and up to two missed trypsin cleavages.
    c. Oxidation of methionine set as dynamic (variable) modification.
    d. Carbamidomethylation of cysteine as static (fixed) modification
    e. False discovery rate below 0.01 at peptide and protein level, using a target decoy approach.
7. Label-free proteins are quantified using the Minora Feature Detector based on the intensity of precursor ions (peak height) of unique and razor peptides.
8. Normalize intensities between samples using total peptide amounts. Median protein abundance ratios are calculated after pairwise comparison of control and infected samples.

9. Calculate adjusted P-values for protein ratios by background-based t-test and adjust using Benjamini-Hochberg correction for false discovery rate.
10. Transfer the data to Excel and filter protein abundance changes using suitable cutoffs. Here used: Proteins detected in at least 2 replicates in infected and 2 replicates in control cultures, > 1 peptide, High Protein FDR confidence ratio. Contaminants are filtered out.
11. Visualize the results in the form of a volcano plot using, e.g., VolcaNoseR2[11], representing log2 (abundance ratio) versus the associated -log10(adjusted p-values). Changes in abundance are considered significant with the following parameters: A log2 (abundance ratio) cutoff of ±1 and adjusted p-value of <0.05.
12. Results are also presented as a bar graph of the proteins plotted against their respective log2 (abundance ratio).

Literature

1. Rasko, D.A.; Rosovitz, M.J.; Myers, G.S.; Mongodin, E.F.; Fricke, W.F.; Gajer, P.; Crabtree, J.; Sebaihia, M.; Thomson, N.R.; Chaudhuri, R.; et al. The pangenome structure of Escherichia coli: comparative genomic analysis of E. coli commensal and pathogenic isolates. *J Bacteriol* **2008**, *190*, 6881-6893, doi:10.1128/jb.00619-08.
2. J, M. A multipurpose cloning system based on the single-stranded DNA bacteriophage M13. *Recombinant DNA Technical Bulletin* **1979**, *2*, 6.
3. Knabben, I.; Regestein, L.; Marquering, F.; Steinbusch, S.; Lara, A.R.; Büchs, J. High cell-density processes in batch mode of a genetically engineered *Escherichia col*i strain with minimized overflow metabolism using a pressurized bioreactor. *Journal of Biotechnology* **2010**, *150*, 73-79, doi:10.1016/j.jbiotec.2010.07.006.
4. Moraru, C. VirClust-A tool for hierarchical clustering, core protein detection and annotation of (prokaryotic) viruses. *Viruses* **2023**, *15*, doi:10.3390/v15041007.
5. Moraru, C.; Varsani, A.; Kropinski, A.M. VIRIDIC—A novel tool to calculate the intergenomic similarities of prokaryote-infecting viruses. *Viruses* **2020**, *12*, 1268, doi:10.3390/v12111268.
6. Garneau, J.R.; Depardieu, F.; Fortier, L.-C.; Bikard, D.; Monot, M. PhageTerm: a tool for fast and accurate determination of phage termini and packaging mechanism using next-generation sequencing data. *Sci Rep* **2017**, *7*, 8292, doi:10.1038/s41598-017-07910-5.
7. Schopper, S.; Kahraman, A.; Leuenberger, P.; Feng, Y.; Piazza, I.; Müller, O.; Boersema, P.J.; Picotti, P. Measuring protein structural changes on a proteome-wide scale using limited proteolysis-coupled mass spectrometry. *Nat Protoc* **2017**, *12*, 2391-2410, doi:10.1038/nprot.2017.100.
8. Wright, B.W.; Logel, D.Y.; Mirzai, M.; Pascovici, D.; Molloy, M.P.; Jaschke, P.R. Proteomic and transcriptomic analysis of *Microviridae* φX174 infection reveals broad upregulation of host *Escherichia coli* membrane damage and heat shock responses. *mSystems* **2021**, *6*, e00046-00021, doi:10.1128/mSystems.00046-21.
9. Howard-Varona, C.; Hargreaves, K.R.; Solonenko, N.E.; Markillie, L.M.; White, R.A., 3rd; Brewer, H.M.; Ansong, C.; Orr, G.; Adkins, J.N.; Sullivan, M.B. Multiple mechanisms drive phage infection efficiency in nearly identical hosts. *ISME J* **2018**, *12*, 1605-1618, doi:10.1038/s41396-018-0099-8.

10. Klaes, S.; Madan, S.; Deobald, D.; Cooper, M.; Adrian, L. GroEL-proteotyping of bacterial communities using tandem mass spectrometry. *International Journal of Molecular Sciences* **2023**, *24*, 15692.
11. Goedhart, J.; Luijsterburg, M.S. VolcaNoseR is a web app for creating, exploring, labeling and sharing volcano plots. *Sci Rep* **2020**, *10*, 20560, doi:10.1038/s41598-020-76603-3.
12. Kropinski, A.M.; Mazzocco, A.; Waddell, T.E.; Lingohr, E.; Johnson, R.P. Enumeration of bacteriophages by double agar overlay plaque assay. *Methods Mol Biol* **2009**, *501*, 69-76, doi:10.1007/978-1-60327-164-6_7.
